# White to beige conversion in PDE3B KO adipose tissue through activation of AMPK signaling and mitochondrial function

**DOI:** 10.1038/srep40445

**Published:** 2017-01-13

**Authors:** Youn Wook Chung, Faiyaz Ahmad, Yan Tang, Steven C. Hockman, Hyun Jung Kee, Karin Berger, Emilia Guirguis, Young Hun Choi, Dan M. Schimel, Angel M. Aponte, Sunhee Park, Eva Degerman, Vincent C. Manganiello

**Affiliations:** 1 Cardiovascular and Pulmonary Branch (CPB), National Heart, Lung, and Blood Institute (NHLBI), National Institutes of Health (NIH), Bethesda, Maryland, 20892, USA; 2 Severance Integrative Research Institute for Cerebral and Cardiovascular Diseases (SIRIC), Yonsei University College of Medicine, Seoul, 03722, Korea; 3 Department of Surgery, Yonsei University College of Medicine, Seoul, 03722, Korea; 4 Lund University Diabetes Center, Department of Experimental Medical Sciences, Lund University, S-221 84 Lund, Sweden; 5 NIH MRI Research Facility, NIH, Bethesda, Maryland, 20892, USA; 6 Proteomics Core Facility, NHLBI, NIH, Bethesda, Maryland, 20892, USA

## Abstract

Understanding mechanisms by which a population of beige adipocytes is increased in white adipose tissue (WAT) reflects a potential strategy in the fight against obesity and diabetes. Cyclic adenosine monophosphate (cAMP) is very important in the development of the beige phenotype and activation of its thermogenic program. To study effects of cyclic nucleotides on energy homeostatic mechanisms, mice were generated by targeted inactivation of *cyclic nucleotide phosphodiesterase 3b (Pde3b)* gene, which encodes PDE3B, an enzyme that catalyzes hydrolysis of cAMP and cGMP and is highly expressed in tissues that regulate energy homeostasis, including adipose tissue, liver, and pancreas. In epididymal white adipose tissue (eWAT) of PDE3B KO mice on a SvJ129 background, cAMP/protein kinase A (PKA) and AMP-activated protein kinase (AMPK) signaling pathways are activated, resulting in “browning” phenotype, with a smaller increases in body weight under high-fat diet, smaller fat deposits, increased β-oxidation of fatty acids (FAO) and oxygen consumption. Results reported here suggest that PDE3B and/or its downstream signaling partners might be important regulators of energy metabolism in adipose tissue, and potential therapeutic targets for treating obesity, diabetes and their associated metabolic disorders.

Obesity is a major risk factor for type 2 diabetes and cardiovascular disease. White adipose tissue (WAT), a highly regulated and dynamic secretory organ, affects body fat and energy utilization through storage and turnover/hydrolysis of triglycerides. In addition, via production of endocrine factors, adipocytokines, and lipids, WAT regulates and integrates important physiological pathways, including satiety, energy utilization, glucose sensitivity, insulin sensitivity, and inflammation^1^. WAT, however, also contributes to metabolic dysregulation that characterizes insulin resistance and obesity-related metabolic and cardiovascular complications^2^,^3^.

Brown adipose tissue (BAT), enriched in mitochondria, regulates adaptive thermogenesis in small rodents and mammalian newborns. Brown adipocytes express mitochondria uncoupling protein 1 (UCP1), which shunts energy derived during mitochondrial β-oxidation of fatty acids (FAO) from ATP formation to thermogenesis^4^,^5^. In general, induction/activation of BAT in rodents is associated with decreased adiposity, improved responsiveness to insulin, and reduced serum free fatty acids (FFA), i.e., a “lean” phenotype which may confer protection against diabetes, obesity, and their metabolic sequelae^6^,^7^,^8^,^9^. Although it is not essential for BAT differentiation, PPARγ co-activator 1 alpha (PGC-1α) is a critical transcriptional activator of cAMP-mediated mitochondrial biogenesis and induction of the thermogenic program^10^. Transfection of cultured human white adipocytes with PGC-1α induced expression of UCP1 and mitochondrial proteins, and increased FAO^11^. Furthermore, PGC-1α-responsive genes involved in FAO and oxidative phosphorylation are upregulated by thiazolidinediones (TZDs)^12^,^13^, and are downregulated in skeletal muscle and adipose tissue in insulin-resistant states^14^,^15^,^16^. Recent studies have demonstrated the presence of substantial amounts of active BAT in adult human WAT depots, and the amount of BAT inversely correlated with BMI (body-mass index), suggesting that human BAT may play a role in regulation of human obesity and energy homeostasis^17^,^18^,^19^. Since white adipocytes and “constitutive” brown adipocytes (e.g., in interscapular brown-fat depots) develop from different precursors and genetic lineages, and since constitutive brown adipocytes and ectopic brown adipocytes (e.g., arising in WAT depots) exhibit distinct but overlapping patterns of gene expression^20^,^21^, the inducible, ectopic “brown-like” cells are referred to as “beige or brite” adipocytes^22^,^23^. Thus, understanding mechanisms (and, thereby, identifying possible drug targets) whereby beige adipocytes arise in WAT reflects a potential strategy in the fight against obesity and diabetes^8^,^9^,^24^,^25^.

cAMP/PKA signaling pathways play critical roles in differentiation of WAT and BAT, and regulation of energy homeostasis^25^. More recently, cardiac natriuretic peptides were also found to induce “browning”, mediated by activation of guanylyl cyclase and cGMP-signaling and activation of PKG and p38 MAPK^26^. Via upregulation of expression of PGC-1α and other genes, cAMP/PKA-signaling increases mitochondrial biogenesis and modulates differentiation of BAT and induction of its thermogenic program^27^. Inhibition of PDE3B by endogenous cGMP might be involved in the reported stimulatory effects of NO and cGMP on BAT differentiation^28^,^29^. In adipocytes, insulin-induced activation of PDE3B is involved in the inhibition of cAMP-stimulated lipolysis by insulin. PDE3B also seems to be important in effects of insulin on glucose uptake and lipogenesis in adipocytes, and in regulation of AMPK activity^30^,^31^,^32^.

Current data suggest that in WAT, PR domain containing 16 (PRDM16) protein is a critical determinant of ectopic “browning”, via its simultaneous induction of beige and suppression of WAT genes^33^,^34^,^35^. PRDM16 interacts with, and strongly coactivates transcription factors, including PGC-1α and PPARα, to induce browning in WAT depots^20^,^21^,^35^,^36^. Via interaction with repressors c-terminal binding protein-1 (CtBP-1) and CtBP-2, PRDM16 inhibits expression of WAT genes^37^. In response to cAMP, PGC-1α is the dominant regulator of mitochondrial biogenesis and thermogenic and oxidative metabolic pathways^38^,^39^. The coactivator function of PGC-1α in modulating gene expression seems to be regulated, in a feedback fashion, by leucine-rich protein 130 (LRP130), a PGC-1α-inducible factor^40^.

AMP-activated protein kinase (AMPK), a sensor or gauge of cellular energy, also plays a critical role in modulating energy homeostasis by regulation of critical metabolic and signaling pathways, e.g., PGC-1α expression and activation, mitochondrial biogenesis, enzymes involved in FAO, via phosphorylation of key downstream effectors and/or by regulation of gene expression^41^,^42^. In adipocytes, AMPK can be activated by cAMP^32^,^43^, perhaps via the increase in the AMP:ATP ratio due, at least in part, to activation of fatty acids released during lipolysis^44^,^45^. AMPK may be a critical mediator of many of the beneficial metabolic effects of the polyphenol resveratrol, which can act as a cAMP phosphodiesterase inhibitor^46^, and the antidiabetic drugs, metformin and TZDs^47^. Thus, AMPK is considered as a potential target to enhance exercise endurance^48^, and to treat obesity-related disorders^49^,^50^.

In this report, we show that targeted inactivation of the murine SvJ129 *Pde3b* gene was associated with activation of cAMP/PKA and AMPK signaling pathways, the integration of which resulted in “browning” of KO eWAT, with increased expression of genes and proteins related to mitochondrial biogenesis and function, thermogenesis, and energy dissipation, including PRDM16, LRP130, PGC-1α, SIRT3, UCP1, ELOVL3, PPARα, and the enzymatic machinery for FAO. Results reported here suggest that PDE3B and/or its downstream signaling partners might be important regulators of energy metabolism and inflammation in adipose tissue, and potential therapeutic targets for treating obesity, diabetes and their associated metabolic disorders.

## Results

### Reduced weight gain by inhibiting PDE3B

**Figure 1:**
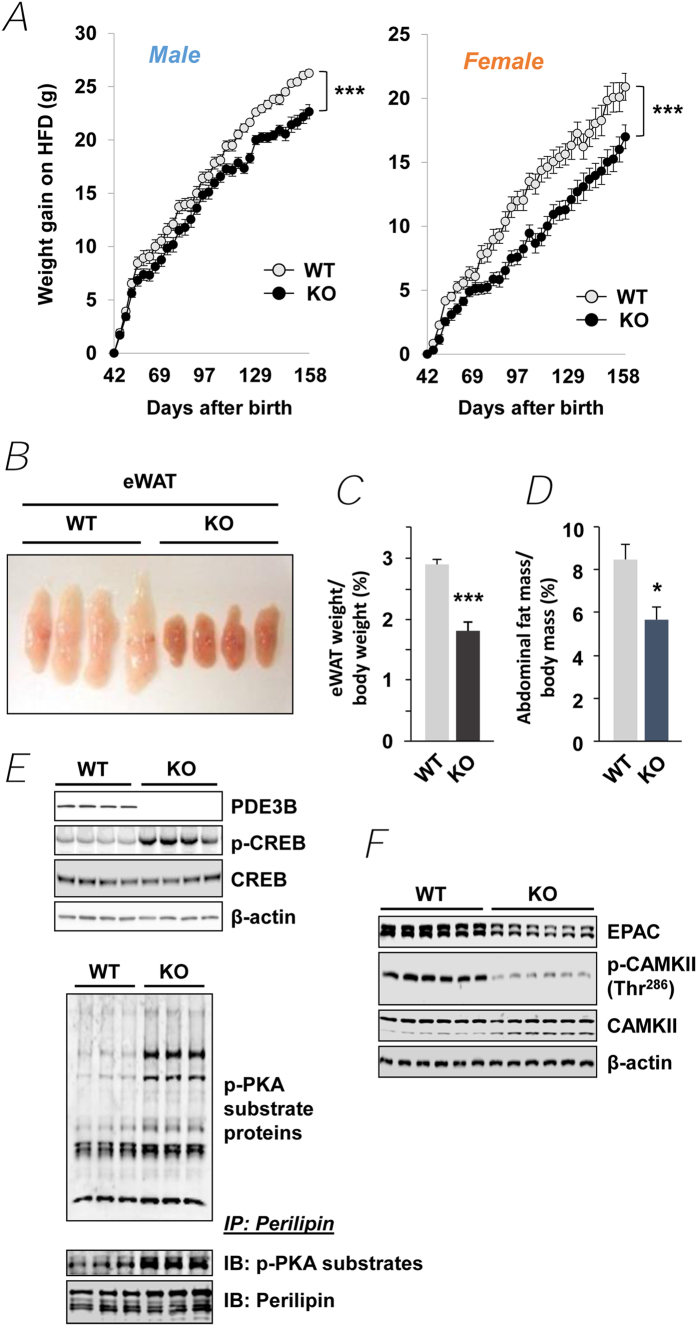
PDE3B KO mice are protected from obesity. (***A***) Weight gain in male and female PDE3B KO mice compared to WT under high-fat diets. Males (WT, *n* = 9; PDE3B KO, *n* = 9) and females (WT, *n* = 6; PDE3B KO, *n* = 9). (***B***) Smaller and brownish epididymal fat pads in PDE3B KO male mice. (***C***) Percent of eWAT weight to body weight (WT, *n* = 66; PDE3B KO, *n* = 46). (***D***) Abdominal fat mass of WT and PDE3B KO analyzed by Micro CT scanning; *n* = 4 mice per each group. (***E***) Immunoblots of PDE3B, CREB, and phosphorylated CREB levels (*Upper*); phosphorylated PKA substrate protein levels in whole lysates (*Middle*); and immunoprecipitates of perilipin in WT and PDE3B KO eWAT (*Lower*). (***F***) Immunoblots of EPAC, CAMKII and phosphorylated CAMKII levels in WT and PDE3B KO eWAT. Data are presented as mean ± SEM. ^***^p < 0.001, ^*^p < 0.05 *vs.* WT.

PDE3B KO mice, which were generated as described in *Methods*, both male and female, gained less weight than wild type (WT) mice during high fat feeding from 40 to 160 days after birth (Fig. 1A). KO eWAT fat pads were smaller than WT pads and “brownish” in color (Fig. 1B), and the percentage of eWAT weight relative to body weight was significantly reduced in KO mice, as compared to WT mice (Fig. 1C). Micro computed tomography (CT) scanning showed that the percentage of abdominal adipose tissue was significantly reduced in KO mice (WT, 8.5 ± 1.4%; KO, 5.7 ± 1.2%) (Fig. 1D). The percentage of gonadal fat weight relative to body weight was also reduced in both HE and KO male and female littermates compared to their WT littermates (Fig. S1). Similar to these results, we previously reported that eWAT mass and adipocyte size were decreased in age-matched male KO mice compared to WT, although body weight was increased in KO mice^31^. As seen in Fig. 1E, although immunoreactive cAMP response element-binding protein (CREB) was not increased in extracts from KO eWAT, phosphorylation of CREB as well as of other substrates of activated PKA, including perilipin and liver kinase B1 (LKB1), an upstream activating kinase of AMPK^51^, were increased. The reduction in immunoreactive exchange nucleotide protein activated by cAMP 1 (Epac1) and phospho-calcium/calmodulin activated protein kinase (CAMK) in KO eWAT (Fig. 1F) suggests that Epac-induced activation of CAMK may not be important in activation of AMPK in KO eWAT^52^,^53^,^54^.

### Development of the beige phenotype in PDE3B KO eWAT, with increased mitochondrial biogenesis and induction of its thermogenic program

**Figure 2:**
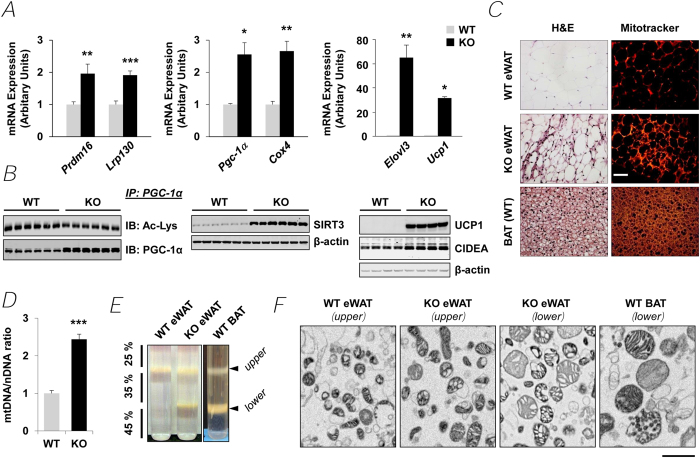
PDE3B KO epididymal fat shows WAT-to-beige phenotypic conversion. (***A***) Quantitative PCR (qPCR) analysis of browning genes in eWAT of WT and PDE3B KO mice. (**B**) Immunoblot analysis of homogenates from WT and PDE3B KO eWAT and immuno-precipitates of PGC-1α. (***C***) Hematoxylin and Eosin (H&E) staining and MitoTracker staining from WT and PDE3B KO eWAT and WT interscapular BAT. Images are shown at 400x magnification. Scale bar, 50 μm. (***D***) Mitochondrial-encoded DNA (*mtDNA*) and nuclear-encoded DNA (*nDNA*) content were presented as a ratio of mtDNA/nDNA with the WT ratio as 1. *n* = 7 mice per each group. (***E***) Mitochondrial fractions (*upper* and *lower* layers) were isolated from eWAT of WT and PDE3B KO mice and interscapular WT BAT via centrifugation in discontinuous sucrose gradients (25%, 35% and 45%). (***F***) Mitochondrial fractions were examined by electron microscopy. Scale bar, 1 micron. Data are presented as mean ± SEM. ^***^p < 0.001, ^**^p < 0.01, ^*^p < 0.05 *vs.* WT.

Gene expression of the three critical coactivators of browning, PRDM16^33^, LRP130^40^,^55^ and PGC-1α^56^ were increased more than 2-fold in KO eWAT (Fig. 2A), whereas expression of two suppressors, retinoblastoma protein 1 (Rb1) and p107^57^,^58^, were decreased (Fig. S2A). Western blots demonstrated that protein expression of Rb1 or p107 was decreased, and that inhibitory phosphorylation of Rb1 at Ser^780^ was increased (Fig. S2B). Gene expression of two other WAT corepressors, CtBP^37^ and nuclear receptor co-repressor (NCoR)^59^,^60^, were also significantly decreased (Fig. S2A). In KO eWAT, activated (deacylated) PGC-1α protein and dramatically increased NAD^+^-dependent protein deacetylase 3 (SIRT3) protein were detected (Fig. 2B), PGC-1α being a key regulator in browning and in mitochondrial biogenesis^25^. PGC-1α regulates transcription of the Sirt3 gene; SIRT3, in turn, regulates effects of cAMP (and PGC-1α) on expression of BAT thermogenic genes, including UCP1, and thus may be important in development of the beige phenotype^21^,^61^. mRNA for nuclear receptor binding factor 1 (Nrbf1)^15^, a transcription factor regulating expression of mitochondrial respiratory proteins, was significantly increased (>2 fold) in KO eWAT, as were mRNAs for β1 and β3 adrenergic receptors (Adrb1 and Adrb3) (Fig. S2A) and ADRB3 protein (Fig. S2B), whose activation is important for function and recruitment of beige^21^ and BAT^4^,^25^,^62^. Expression of genes related to mitochondrial respiration (e.g. Cox4, cytochrome c oxidase subunit 4 isoform 1) (Fig. 2A) and immunoreactive mitochondrial COX1 (cytochrome c oxidase subunit 1) protein (Fig. S2B) were also increased in KO eWAT. Transient receptor potential vanilloid 4 (TRPV4) is a negative regulator of PGC-1α and UCP1 and a positive regulator of expression of proinflammatory genes^63^. Thus, the reduced protein expression of TRPV4 in KO eWAT may be important in recruitment/differentiation of beige adipocytes, i.e., increased expression of PGC-1α and induction of UCP1^64^. The expression of several genes crucial to the beige fat thermogenic program was upregulated in KO eWAT. Elongase of very long chain fatty acid-like 3 (*Elovl3)*, which is detected only in BAT and liver and is implicated in the thermogenic function of BAT^65^, was markedly increased in PDE3B KO eWAT (Fig. 2A). *Ucp1*, expressed almost exclusively in BAT mitochondria, was increased 31-fold (Fig. 2A). Cell death-inducing DNA fragmentation factor, alpha subunit-like effector A (*Cidea*), encoding a mitochondrial protein important in regulation of energy balance and adiposity, in part by suppressing UCP1 activity^66^, was increased 11-fold (Fig. S2A), suggesting coordinated control of energy metabolism in PDE3B KO eWAT. Type2 deiodinase (*Dio2*), induced by cAMP and essential for thyroid-sympathetic synergism in thermal homeostasis^67^,^68^, was also increased 3-fold (Fig. S2A). Western blot analysis demonstrated that KO eWAT expressed the BAT-specific marker proteins UCP1 and CIDEA (Fig. 2B).

As seen in Fig. 2C, hematoxylin and eosin (H&E) staining of eWAT from WT and KO littermates and of WT interscapular BAT showed that cytoplasmic spaces in KO eWAT were thicker than WT eWAT, and more similar to that of BAT. Mitotracker staining indicated that mitochondrial density was increased in KO mice. Confocal microscopy image also showed that compared to WT eWAT, mitochondrial density was increased in KO eWAT adipocytes, as were markers for vascular endothelium (CD31) and vascular smooth muscle actin (SMA) (Fig. S3). Mitochondrial staining and vascular markers in KO eWAT resembled interscapular BAT. Taken together, eWAT of KO mice acquired characteristics of BAT, including more blood vessels and mitochondria, which give it a “brownish” color^4^,^25^. Consistent with the observed increases in mitochondrial staining, KO eWAT mitochondrial DNA was increased (Fig. 2D). During purification of mitochondria via centrifugation in discontinuous sucrose gradients, WT interscapular BAT mitochondria sedimented as upper and lower bands, with the latter predominant. To a large extent, however, WT eWAT mitochondria sedimented as upper bands. KO eWAT mitochondria, on the other hand, sedimented in upper and lower bands”, similar to interscapular BAT mitochondria (Fig. 2E). As seen in electron micrographs of purified mitochondria (Fig. 2F), mitochondria from KO eWAT were larger than those from WT eWAT.

### Increased energy dissipation by activation of AMPK signaling in PDE3B KO eWAT

**Figure 3:**
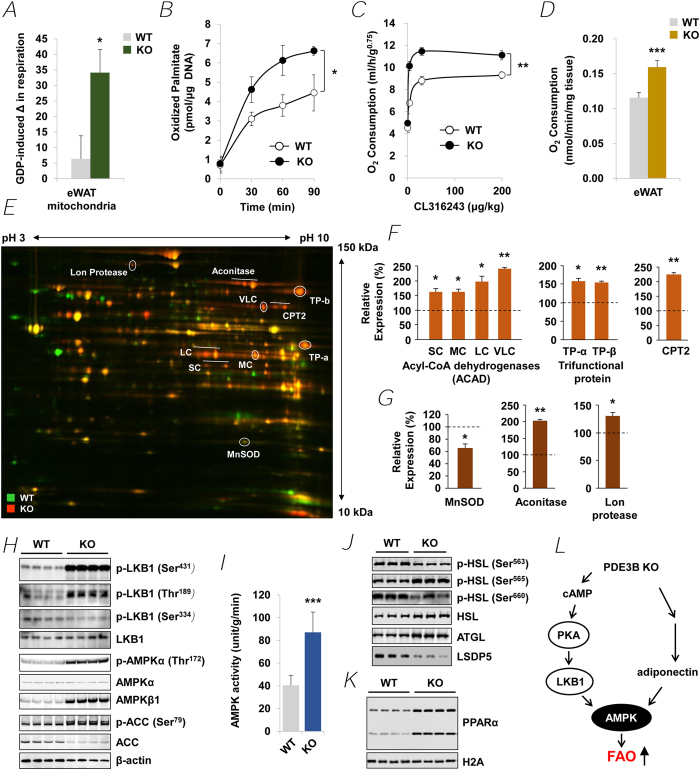
Mice lacking PDE3B have increased metabolic rate due to an activation of AMPK signaling pathways. (***A***) GDP-sensitive proton leak (uncoupled respiration due to UCP1) in eWAT mitochondria from WT and PDE3B KO mice. *n* = 3 mice per each group. (***B***) Oxidation of [9,10(n)-^3^H]Palmitic acid (pmol oxidized/μg DNA) in adipocytes from WT and PDE3B KO eWAT at indicated time points (0, 30, 60 and 90 min). Values are presented as mean of three independent experiments. (***C***) Whole body O_2_ consumption in intact WT and PDE3B KO mice after intraperitoneal administration of indicated concentrations of CL316243 (CL) (0, 4, 30, or 200 μg/kg); *n* = 5 mice per each group. (***D***) *In vitro* O_2_ consumption in fragments of eWAT from WT and PDE3B KO mice; *n* = 7 mice per each group. (***E***) Two-dimentional difference gel electrophoresis (DIGE) of eWAT mitochondrial fractions from WT (*n* = 10) and PDE3B KO (*n* = 5) mice. The gel image is representative of three independent experiments. (***F*** and ***G***) Quantitative proteomics analysis of DIGE. Seven mitochondrial FAO-related enzymes (***F***) and three indicators of mitochondrial oxidative stress (***G***); *n* = 3 DIGE gels. (***H***) Immunoblots showing LKB1, phosphorylated LKB1, AMPK, phosphorylated AMPK, ACC and phosphorylated ACC levels in WT and PDE3B KO eWAT. (***I***) AMPK activity in PEG-precipitated fractions of eWAT homogenates; *n* = 8 per each group. (***J***) Immunoblots showing HSL, phosphorylated HSL, ATGL and LSDP5 levels in WT and PDE3B KO eWAT. (*K*) An immunoblot showing PPARα levels in nuclear extracts from WT and PDE3B KO eWAT. (***L***) Model for *Pde3b* deletion-induced browning. In KO eWAT, cAMP-dependent PKA and AMPK pathway axis plays an important role in the regulation of the beige phenotype, energy homeostasis, FAO, and lipid metabolism. Data are presented as mean ± SEM. ^***^p < 0.001, ^**^p < 0.01, ^*^p < 0.05 *vs.* WT.

As might be expected from the marked increase in UCP1 in PDE3B KO eWAT, mitochondrial respiration was uncoupled in KO mitochondria, compared to WT (Fig. 3A). FAO was significantly increased in isolated adipocytes from PDE3B KO mice compared to WT (Fig. 3B). Compared to WT, oxygen consumption was significantly increased in intact PDE3B KO mice following intraperitoneal (IP) injection of the β3-specific adrenergic receptor agonist, CL316243 (CL) (Fig. 3C), and *in vitro* in isolated eWAT (Fig. 3D) fragments from PDE3B KO mice. These results are consistent with PDE3B KO eWAT assuming phenotypic characteristics of beige adipose depots, i.e., an increase in mitochondrial biogenesis, with increased energy dissipation and FAO^21^,^69^.

As seen in Fig. 3E, to identify differences in WT and KO eWAT mitochondrial proteomes, WT and KO eWAT mitochondrial preparations analyzed by two-dimensional difference gel electrophoresis (DIGE) as described in *SI Materials and Methods*. After coomassie blue staining of DIGE gels (WT and KO mitochondria combined), a total of 145 protein spots of the eWAT mitochondrial proteome were identified by matrix-assisted laser desorption ionization (MALDI)-time-of-flight (TOF) MS/MS (Fig. S4). Based on image analysis of Cy3/Cy5 (KO/WT) fluorescence of three independent DIGE gels using Progenesis Discovery software (NonLinear Dynamics, Durham, NC), relative differences in expression of MS/MS-identified proteins in WT and KO eWAT mitochondria were calculated and represented as bar graphs (Fig. 3F and G) and listed in Table S2. As seen in Fig. 3F, DIGE results indicated that mitochondrial FAO-related proteins were significantly increased in KO eWAT mitochondria, including four isotypes of acyl-CoA dehydrogenase (ACAD), carnitine palmitoyltransferase 2 (CPT2), and α and β subunits of trifunctional protein (TP), all of which increased more than 1.5-fold. DIGE results also demonstrated that KO eWAT mitochondria were under less oxidative stress, in that expression of manganese superoxide dismutase (MnSOD), a mitochondrial antioxidant enzyme^70^, was lower in KO than WT (Fig. 3G). Higher expression of the Lon protease and its preferential substrate aconitase in mitochondria from KO eWAT indicated that they might be less damaged by oxidative stress, since oxidatively modified proteins are degradated by the Lon protease in the mitochondrial matrix^71^. The expression levels of mitochondria complexes I, II, III and IV were not significantly changed in KO mitochondria compared with WT (Table S2). To determine whether changes in mitochondria proteome were limited to adipose tissue, mitochondria isolated from WT and KO liver, spleen, heart and brain were analyzed. Although some spots demonstrated differential expression in each tissue, the overall differential changes in these tissues were fewer than those in eWAT mitochondrial proteomes from WT and PDE3B KO mice (Fig. S5).

In KO eWAT, LKB1 was activated, as evidenced by its auto-phosphorylation at Thr^189^ and phosphorylation at Ser^431^ (most likely via activated PKA) (Fig. 3H)^72^,^73^. Although immunoreactive AMPKα was not increased in KO eWAT, phosphorylation of AMPKα at Thr^172^, required for AMPK activation^41^, was significantly increased, as was AMPK enzymatic activity in PEG-precipitated fractions from KO eWAT (WT, 40.5 ± 8.7 unit/g/min; KO, 87.0 ± 17.8 unit/g/min) (Fig. 3I). Protein expression of AMPK β1 (a targeting subunit) was also increased in KO eWAT. Activation of AMPK was associated with decreased expression of acetyl-CoA carboxylase (ACC) protein and increased phosphorylation of ACC at Ser^79^ (Fig. 3H). Phosphorylation of ACC is thought to be critical in AMPK-induced reduction in malonyl CoA and activation of mitochondrial FAO^74^,^75^. Activation of FAO could be related to cAMP/PKA-induced phosphorylation/activation of SIRT1, leading to deacetylation/activation of PGC-1α and increased FAO, independent of changes in NAD^+ ^76. In addition, activation of AMPK in KO eWAT was associated with increased expression and phosphorylation/activation of endothelial nitric oxide synthase (eNOS) (Fig. S2C), an important regulator of mitochondrial biogenesis^77^. Expression of other proteins important in cAMP-signaling, i.e, PKA regulatory subunit II (PKA-RII) (Fig. S2C) and proteins involved in regulation of lipolysis, i.e., perilipin (Fig. 1E), adipose triglyceride lipase (ATGL) and hormone-sensitive lipase (HSL), were increased in KO eWAT, whereas another lipid droplet protein, lipid storage droplet protein-5 (LSDP5), was markedly decreased (Fig. 3J). As seen in Fig. 3J, in KO eWAT, as compared to WT eWAT, HSL was relatively more heavily phosphorylated at Ser^565^, an AMPK-sensitive, inhibitory site^78^, than at Ser^563^, the PKA-sensitive activating site, consistent with previous findings in PDE3B KO mice, which indicated that, in the fed state, serum FFA were not elevated in KO mice^31^. On the other hand, AMPK has also been reported to phosphorylate/activate ATGL, which is critical in development of BAT and, perhaps, as a source of fatty acids for FAO^79^. Activation of AMPK and the subsequent phosphorylation (inactivation) of ACC (Fig. 3H) is important in regulation of FAO and of systemic insulin sensitivity^41^. As seen in Fig. S2A, expression of mRNAs for PPARα, a master nuclear transcriptional regulator for FAO genes was increased more than 5-fold in PDE3B KO eWAT. PPARα protein, not detectable or barely detectable in WT eWAT^25^, was also increased in nuclear extracts from KO eWAT (Fig. 3K). Consistent with the result of DIGE, Western blotting of eWAT homogenates demonstrated that protein expression of two key enzymes important for fatty acids uptake into mitochondria, CPT1 and CPT2, was also increased in KO eWAT (Fig. S2D).

### Role for PDE3B in cAMP/PKA- and AMPK-signaling in 3T3-L1 adipocytes

**Figure 4:**
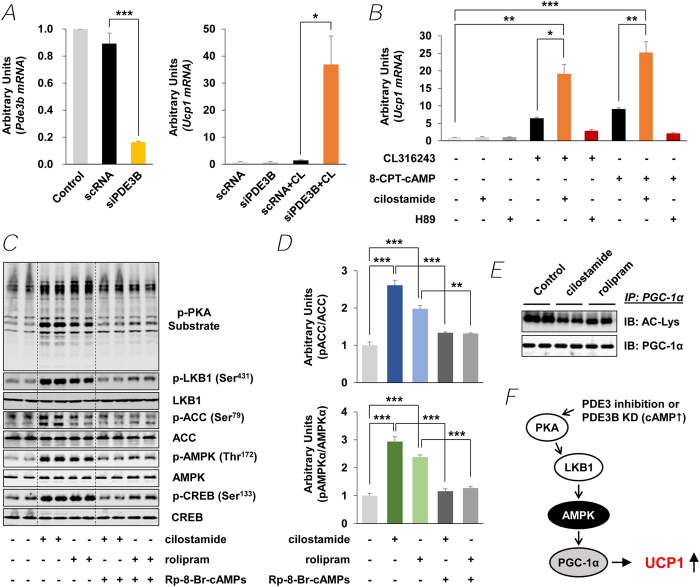
Knockdown or chemical inhibition of PDE3B increases UCP1 expression by AMPK activation in 3T3-L1 adipocytes. (***A***) qPCR analysis of *Pde3b* and *Ucp1* transcripts after PDE3B knockdown in 3T3-L1 adipocytes; *n* = 3 experiments, duplicate assays. sc RNA, scrambled RNA; siPDE3B, PDE3B siRNA. (***B***) qPCR analysis of *Ucp1* after PDE3 inhibition by cilostamide (10 μM) or PKA inhibition by H89 (10 μM) in 3T3-L1 adipocytes. CL316243 (0.1 μM) or 8-CPT-cAMP (100 μM) were treated for 4 h. (***C***) Immunoblot analysis of 3T3-L1 adipocytes after PDE3 inhibition by cilostamide (10 μM) or rolipram (30 μM). PKA-specific inhibitory cAMP analog (Rp-8-Br-cAMPs) were treated for 15 min. (***D***) Ratio of pACC/ACC (*Upper*) and pAMPK/AMPK (*Lower*) are presented as bar graphs. (***E***) Immunoblot analysis of immunoprecipitates of PGC-1α from 3T3-L1 adipocytes after PDE3 inhibition by cilostamide or rolipram. Blots represent one of three independent experiments. (***F***) Model for PDE3B inactivation-induced up-regulation of UCP1. Data are presented as mean ± SEM. ^***^p < 0.001, ^**^p < 0.01, ^*^p < 0.05.

A regulatory role for PDE3B in induction of UCP1 in PDE3B KO eWAT was confirmed in 3T3-L1 adipocytes. In differentiated adipocytes transfected with PDE3B siRNA, UCP1 mRNA was dramatically increased ~37 fold during incubation with CL compared to control oligonucleotide-transfected cells (Fig. 4A). Incubation of differentiated 3T3-L1 adipocytes with cilostamide, a PDE3 inhibitor, also amplified the expression of UCP1 induced by CL and 8-CPT-cAMP, a cAMP analogue. Furthermore the induction of UCP1 by CL and 8-CPT-cAMP was inhibited by H89, a PKA inhibitor, suggesting a regulatory role for cAMP and PKA (Fig. 4B). As seen in Fig. 4C, inhibition of PDE3B with cilostamide increased phosphorylation of CREB at Ser^133^, AMPKα at Thr^172^, and ACC at Ser^79^, as well as of other unidentified PKA substrates. Rolipram, a PDE4 inhibitor, had a smaller effect on phosphorylation of AMPKα and ACC (Fig. 4C and D), suggesting PDE3B might regulate a distinct signaling pathway(s) that activates AMPK. The PKA inhibitor, Rp-8-Br-cAMPs, inhibited the effects of cilostamide and rolipram on phosphorylation of AMPK and ACC. Effects of cAMP on the activation status of PGC-1α were examined in differentiated 3T3-L1 adipocytes, which were incubated with cilostamide or rolipram. Total solubilized protein (1 mg) from differentiated 3T3-L1 adipocytes were immunoprecipitated with anti-PGC-1α antibody, and then immunoblotted with an anti-acetyl lysine antibody or the PGC-1α antibody, to determine both PGC-1α acetylation at lysine residues (Ac-Lys) and total PGC-1α protein. In contrast to PDE3B KO eWAT (Fig. 2B), expression of PGC-1α did not change in differentiated 3T3-L1 adipocytes treated with the PDE3 inhibitor cilostamide, but PGC-1α was deacetylated (activated) (Fig. 4E). Deacetylation of PGC-1α was also observed in the presence of rolipram.

## Discussion

The phenotypic characteristics of PDE3B KO mice in SvJ129 background are complex, reflecting the role of PDE3B in tissues important for regulating energy homeostasis, i.e. adipose tissue, pancreas, and liver^31^. In KO mice, gonadal adipose tissue mass and eWAT adipocytes were smaller than their WT counterparts. Importantly, as demonstrated here, KO eWAT mice acquired characteristics of a beige phenotype, including changes in morphology, increased expression of genes related to differentiation/recruitment of beige adipocytes, increased mitochondrial biogenesis, increased UCP1 (usually absent in WAT), increased FAO, and increased O_2_ consumption and energy dissipation^21^. Adiponectin, a circulating WAT-specific adipocytokine, may be responsible for some of these phenotypic changes. As reported earlier, serum adiponectin levels, which correlate inversely with percent body fat, were significantly increased in KO mice^31^. Consistent with the phenotype of KO mice described herein, adiponectin has been reported to activate AMPK and FAO in liver and skeletal muscle, and also exert anti-inflammatory actions and cardioprotective effects^80^,^81^. These latter effects of adiponectin may also be related to activation of AMPK, which was reported to increase mitochondrial biogenesis and decrease reactive oxygen species (ROS) in human umbilical vein endothelial cells (HUVECs)^82^. Thus, PDE3B KO mice in SvJ129 background may provide a model for induction of beige adipocytes in WAT, recently postulated as a potential new strategy to combat diabetes and obesity-related diseases^8^,^9^,^24^,^25^.

cAMP is very important in the development of the beige phenotype and activation of its thermogenic program^21^,^22^. As seen in Figs 1E and 3H, in KO eWAT, deletion of PDE3B increased cAMP/PKA signaling (phosphorylation of CREB and other PKA substrates including LKB1), as well as AMPK signaling, the integration of which resulted in upregulation of PGC-1α and PPARα, and induction of PRDM16 and LRP130 and suppression of Rb1, p107, CtBP and NCoR. As outlined in Fig. 3L and Fig. S8, in PDE3B KO eWAT, PRDM16, together with PGC-1α, PPARα, Nrbf-1 and other transcriptional regulators, i.e. CtBP, NCoR, LRP130, Rb1, and p107, initiated a coordinated metabolic program by upregulating PGC-1α, ELOVL3, CIDEA, and DIO2, and critical mitochondrial proteins, including UCP1, CPT2, CACT, ACAD, and phosphorylated ACC, which might be responsible for increased thermogenesis, energy dissipation, O_2_ consumption, and FAO in PDE3B KO eWAT. In 3T3-L1 adipocytes, down-regulation of PDE3B, via pharmacologic inhibition with cilostamide or knockdown with siRNA PDE3B oligonucleotides, increased cAMP/PKA and AMPK signaling resulting in activation of PGC-1α and enhanced induction of UCP1 mRNA in response to CL or 8-CPT-cAMP (Fig. 4B). Thus, we suggest that, as summarized in Figs 4F and S8, PDE3B may regulate a cAMP-sensitive molecular “switch” for “browning” of eWAT^27^. It is important to note that genetic background plays an important role in this PDE3B-regulated cAMP-sensitive WAT to beige phenotypic transition. In contrast to this current work with SvJ129 PDE3B KO mice, where deletion of PDE3B alone was sufficient to induce the beige phenotype in KO eWAT, in C57BL/6 PDE3B KO mice, we reported that administration of the β3 adrenoreceptor agonist, CL induced the beige transition^83^. Although several specific genes are considered as beige adipocyte markers, expression of these genes is quite variable in different types of adipocytes. As shown in Fig. S2E, solute carrier family 27 member 1 (Slc27a1, also known as Fatp1) was increased 2.7-fold and transmembrane 26 (Tmem26) and short stature homeobox-2 (Shox2) were significantly decreased in eWAT of PDE3B KO mice^21^,^84^,^85^,^86^.

Our data (Fig. 3L) support the idea that increased cAMP signaling in KO eWAT leads to activation of AMPK-signaling, perhaps via PKA-mediated phosphorylation/activation of LKB1. A complex between LKB1, mouse protein 25 (MO25), and STRAD (pseudokinase STE-related adaptor protein) has been identified as the major upstream kinase responsible for phosphorylation of AMPK on its activating loop at Thr^172^ 87,88. ATGL, which apparently plays an important role in development of BAT and can be activated by AMPK^79^, is increased in KO eWAT and may generate fatty acids for FAO. AMP, generated during acylation of fatty acids released during lipolysis, binds to and activates phosphorylated AMPK (most likely phosphorylated by phosphorylated/activated LKB1)^44^. AMPK may also be phosphorylated/activated in KO eWAT a part of an autocrine response to increased serum adiponectin released from WAT in KO mice^31^,^45^,^89^. Activated AMPK can phosphorylate\inactivate ACC (Fig. 3H), leading to decreased production of malonyl CoA, which relieves inhibitory constraints on CPT1, a rate-limiting enzyme for activation of FAO (Fig. S2D)^75^. Analysis of isolated eWAT mitochondria by electron microscopic and proteomics techniques, and functional assays indicated that KO eWAT mitochondria were larger than WT and that the proteomic pattern in KO mitochondria was consistent with an increase in FAO-related proteins and decrease in the amount of oxidatively damaged proteins. Thus, as outlined in Fig. S8, not only were transcriptional regulators such as PGC-1α and PPARα and enzymatic machinery for FAO upregulated and negative regulators such as TRPV4 downregulated, but AMPK activity was increased, resulting in activation of FAO. Studies in 3T3-L1 adipocytes also indicated that cilostamide (PDE3 inhibitor) was more effective than rolipram (PDE4 inhibitor) in increasing phosphorylation of AMPK and ACC, suggesting that PDE3B regulated a cAMP “pool” important in regulation of AMPK and, perhaps, SIRT3 (Fig. 2B).

A recent report^46^ has suggested that many of the beneficial metabolic effects of the polyphenol resveratrol may be mediated by inhibition of cAMP PDEs, and, as shown in Table 1, the phenotypic changes in KO eWAT that result in activation of cAMP/PKA and AMPK signaling and increased mitochondrial biogenesis, FAO, and energy dissipation are quite similar to effects of administration of rosiglitazone to ob/ob mice^90^ or resveratrol to rodents^46^. This suggests that a switch to the beige phenotype and increased FAO in WAT may mediate some of the beneficial effects of insulin-sensitizing drugs such as rosiglitazone and resveratrol. The concept regarding salutary effects of acquisition of beige characteristics by WAT and enhanced energy dissipation is consistent with reports indicating that PGC-1α-responsive genes related to oxidative phosphorylation are coordinately down regulated in human diabetes^14^,^15^,^16^.

PDE3B KO mice also exhibited signs of insulin resistance, with reduced glucose removal in response to insulin^31^. The liver is most likely the primary site, since insulin-induced suppression of glucose production, but not insulin-stimulated glucose uptake, was blocked in PDE3B KO mice^31^. Furthermore, in KO liver, cAMP was increased and, especially during a 6 h fast, PGC-1α expression was also increased as were key gluconeogenic enzymes, Trible-3, and inflammation/stress-related signaling molecules^31^. Thus, alterations in cAMP and PGC-1α may play critical roles in phenotypic characteristics of KO liver and eWAT. In adipose tissue, PGC-1α apparently regulates browning, adaptive thermogenesis, and mitochondrial function, biogenesis and respiration^27^. In liver, however, PGC-1α may also promote insulin resistance via co-activation of PPARα and induction of Trible-3^91^. Although PDE3B KO mice show signs of insulin resistance^31^, they are lean and not diabetic, most likely due to development of the “fat-burning” beige phenotype and other characteristics of healthy KO eWAT discussed in this report, which compensate for the insulin resistance. In PDE3B KO mice, changes in adiponectin as well as the increase in insulin secretion in response to glucose, glucagon-like peptide1 and CL^31^, and the presence of smaller adipocytes and gonadal fat depots, as well as enhanced energy dissipation, might represent additional mechanisms that maintain or improve sensitivity to insulin, and/or prevent more marked insulin resistance and glucose intolerance. In this study we focused on eWAT since initially we noted pronounced changes in this depot. This was a little bit unexpected since most mouse work has focused on the subcutaneous inguinal white adipose tissue (iWAT), and its dynamics including feed/fasting, inflammation, and browning. Thus much of the systemic phenotype observed in PDE3B KO mice may still be due to changes in the iWAT depot.

In summary, PDE3B is an important regulator of lipid metabolism, adiposity, and energy status in adipocytes (Fig. S8). PDE3B deficiency resulted in cAMP/PKA- and AMPK-induced increases in respiratory uncoupling and FAO, which could favorably impact energy homeostasis, circulating FFA, and insulin sensitivity. Thus, adipocyte PDE3B, and/or cAMP pathways regulated by PDE3B, may provide new targets for the development of anti-obesity drugs designed to produce beneficial effects by inducing the beige phenotype in WAT.

## Methods

### PDE3B KO mice

PDE3B KO mice were progeny of 7–10 backcrosses of heterozygous (HE) F1 mice with JAX 129/SvJ (pTyr^c-ch^/pTyr^c^) substrain^31^. With primers from *Pde3b* exons 1/2 and 8/9, mRNA amplification in PDE3B KO eWAT was ~5% that of WT. With primers 3′ to exon 9, i.e. exons 9/10 and 15/16, mRNA amplification was ~15% that of WT (Fig. S6A–C). PDE3 enzymatic activity was virtually absent in KO eWAT membrane fractions (Fig. S6E and F, Fig. S7A). There was very little PDE3 activity in WT or KO eWAT cytosol (Fig. S6E and F, Fig. S7F). As seen in Fig. S6, analysis of eWAT PDE3 activity and immunoreactivity after gel filtration chromatography of solubilized membrane fractions and cytosolic fractions indicated that residual PDE3 activity in KO eWAT was related to the presence of PDE3A, not PDE3B, as previously suggested for residual PDE3 activity in PDE3B KO liver^31^. Mice were maintained, and studies performed, in accord with protocols (Protocol H-0024R4) approved by the NHLBI Animal Care and Use Committee. Mice were fed high-fat-diet in the weight gain study, but all other experiments were done on chow diet.

### Statistical Analysis

Data are expressed as mean ± SEM. Student’s t test was used for single variables, one-way ANOVA with Bonferroni post hoc correction was used for multiple comparisons and two-way ANOVA followed by Bonferroni posttests was used for multiple variables using GraphPad Prism 5 software. Values of *p* less than 0.05 were considered to be statistically significant and are presented as *(*p* < 0.05), **(*p* < 0.01), ***(*p* < 0.001).

Further details are given in *SI Materials and Methods*.

## Additional Information

**How to cite this article**: Chung, Y. W. *et al*. White to beige conversion in PDE3B KO adipose tissue through activation of AMPK signaling and mitochondrial function. *Sci. Rep.* **7**, 40445; doi: 10.1038/srep40445 (2017).

**Publisher's note:** Springer Nature remains neutral with regard to jurisdictional claims in published maps and institutional affiliations.

## Supplementary Material

Supplementary Information

## Figures and Tables

**Table 1:** Comparison of effects of anti-diabetes drugs with eWAT phenotype in PDE3B KO mice.

*Phenotypes and Markers*		*PDE3B KO (eWAT)*		*Rosiglitazone (ob/ob)*		*Resveratrol*
**Epididymal Fat Pad**	Fat Mass	↓^31^				↓^92^
	Adipocyte Size	↓^31^		↓		↓^93^
**PKA/AMPK Signaling Pathways**	Phosphorylated-PKA Substrates	↑				↑^94^
	Phosphorylated-CREB	↑				↑^95^
	Phosphorylated-Perilipin	↑				
	Phosphorylated AMPK	↑		↑^96^		↑^46^
	Phosphorylated-ACC	↑		↑^96^		↑^46^
	AMPK Activity	↑		↑^96^		↑^46^,^97^
**Insulin Secretion**		↑^31^				↑^98^
**Mitochondrial Biogenesis**	Mitochondrial Mass	↑		↑		↑
	***Related Genes***	***mRNA***	***Protein***	***mRNA***	***Protein***	
	UCP1	↑	↑	↑^90^		↑^97^
	PGC-1α	↑	↑	↑^90^		↑^97^
	Acetylated-PGC-1α	↓				↓
	COX	↑	↑	↑^90^	↑^90^	↑^99^
	CPT	↑	↑	↑^90^		↑^100^
	ACAD	↑	↑	↑^90^		
	Aconitase		↑	↑^90^		
**FAO**	PPARα	↑		↑^101^		↑^102^
	Isolated Adipocytes	↑		↑		↑^97^
**Oxygen Consumption**	Isolated Adipose Tissue	↑		↑		
	Intact Mice	↑ (+*β3 agonist*)				
	C2C12 Myotubes					↑^46^
