# Supplementary material for: White to beige conversion in PDE3B KO adipose tissue through activation of AMPK signaling and mitochondrial function
Source: Sci Rep. 2017 Jan 13;7:40445. doi: 10.1038/srep40445 (PMC5234021; doi:10.1038/srep40445)
Supplement: Supplementary Information [file srep40445-s1.pdf]

Supplementary Information

White to beige conversion in PDE3B KO adipose tissue through activation of AMPK signaling and mitochondrial function

Youn Wook Chung,[1,2,7,*] Faiyaz Ahmad,[1,7] Yan Tang,[1,7] Steven C. Hockman,[1] Hyun Jung Kee,[3] Karin Berger,[4] Emilia Guirguis,[1] Young Hun Choi,[1] Dan M. Schimel,[5] Angel M. Aponte,[6] Sunhee Park,[1] Eva Degerman,[4] Vincent C. Manganiello,[1]

[1]Cardiovascular and Pulmonary Branch (CPB), National Heart, Lung, and Blood Institute (NHLBI), National Institutes of Health (NIH), Bethesda, Maryland, 20892, USA

[2]Severance Integrative Research Institute for Cerebral and Cardiovascular Diseases (SIRIC),

[3]Department of Surgery,

Yonsei University College of Medicine, Seoul, 03722, Korea

[4]Lund University Diabetes Center, Department of Experimental Medical Sciences, Lund University, S-221 84 Lund, Sweden

[5]NIH MRI Research Facility, NIH, Bethesda, Maryland, 20892, USA

[6]Proteomics Core Facility, NHLBI, NIH, Bethesda, Maryland, 20892, USA

[7]Co-first author

*Correspondence: chungyw@yuhs.ac (Y. W. C.)

Supplementary Materials and Methods

Antibodies

Antibodies for immunoblotting were obtained as follows from specified commercial sources with their catalog numbers in parentheses: from Cell Signaling Technology (Beverly, MA): AMPK-α (2532), AMPK-β (12063), phospho-AMPK-α Thr^{172} (2535), p-AMPK-β-Ser^{108} (4181), ACC (3662), p-ACC-Ser^{79} (3661), ATGL (2138), acetylated-lysine mouse monoclonal antibody (9681), CREB (9197), CAMK$_{II}$ (3362), p-CAMK$_{II}$-Thr^{286} (3361), EPAC1 (4155), fatty acid synthase (3180), H2A (2578), HSL (4107), p-HSL-Ser^{563} (4139), p-HSL-Ser^{565} (4137), p-HSL-Ser^{660} (4126), LKB1 (3047), p-LKB1-Ser^{431} (3482), p-LKB1-Ser^{334} (3055), perilipin (3470), p-PKA substrate (9621), Rb1 (9313), p-Rb-Ser^{780} (8180), SIRT3 (5490), and histone H3 (9717); from Millipore, Inc. (Billerica, MA): p-CREB-Ser^{133} (06-519); from Thermoscientific (Rockford, IL): PPARα (MA1-822), and CIDEA (PA1-84478); from Sigma-Aldrich Corp. (St. Louis, MO): β-actin (A-5441); from Santa Cruz Biotechnology (Santa Cruz, CA): PGC-1α (SC-13067), and β3-AR (SC-50436); from Alpha Diagnostic International (San Antonio, TX): CPT1 (CPT1M11-A), and CPT2 (CPT21A); from BD Biosciences, Inc (San Jose, CA): eNOS (610297), p-eNOS-Ser^{1177} (612393), PKA-RII (610626), PKA-RI (610166), PKA-C (610981), and PP2A (610556); from Assay Biotechnology Company Inc. (Sunnyvale, CA): p-LKB1-Thr^{189} (A-0673); from Protein Tech Group (Chicago, IL): COX1 (Oxphos complex4 Subunit 1) (459600); from Abcam (Cambridge, MA): UCP1 (ab-10983), CD31 (ab-24590), and smooth muscle actin (SMA) (7817); from Novus Biologicals (Littleton, CO): LSDP5 (NB110-60509). Rabbit polyclonal antibodies against mouse PDE3B (GenBank® accession number AAN52086) were generated [1] against peptides corresponding to the CT (C-terminal) domain (amino acids 1076–1095; NASLPQADEIQVIEEADEEE), and the NT (N-terminal) domain (amino acids 2–16; RKDERERDAPAMRSP). Affinity-purified anti-PDE3B-NT and anti-PDE3B-CT antibodies were used for Western blotting.

Real-time quantitative PCR (qPCR) assays

Total RNA was diluted to 10 ng/μl and 100 ng of RNA were subjected (in duplicate) to Real-time quantitative RT-PCR on the HT7900 Sequence Detection System (Applied Biosystems) by using QuantiTect SYBR Green RT-PCR kit (Qiagen) according to manufacturer's protocols. The value of the target gene was normalized by that obtained from cyclophilin A, which served as the internal control. The ratio of the individual normalized value of KO (or WT) mice to the average of normalized values of WT (or KO) mice was calculated and the average was defined as an arbitrary unit. The sequences of primers are listed in ***Table S1***.

Mitochondrial DNA content quantification

Genomic DNA was isolated from eWAT of WT and KO mice using the DNeasy Tissue kit (QIAGEN, Valencia, CA) and analyzed by quantitative PCR analysis using SYBR green (Applied Biosystems, Foster City, CA). The mitochondrial DNA (mtDNA) was assessed using primers for the mitochondrial-encoded gene *Cyt b* (5'-GTG AAC GAT TGC TAG GGC C-3' and 5'-CGA TTC TTC GCT TTC CAC TTC AT-3') and the nuclear DNA (nDNA) was determined by amplifying the nuclear-encoded gene *H19* (5'-GTA CCC ACC TGT CGT CC-3' and 5'-GTC CAC GAG ACC AAT GAC TG-3'). The ratio of mtDNA to nDNA was determined by normalizing *Cyt b* gene copy number to *H19* gene copy number.

High-fat diet studies

Age-matched (2-month old) WT and KO mice were housed two per cage with food and water *ad libitum*. The mice were fed high-fat diets (D12492 Research Diets, NJ) and low-fat diets (D12450B, Research Diets) for 14 weeks. Protein, carbohydrate, and fat contents as a percentage of caloric content were 20, 70, and 10 kcal% for low-fat, and 20, 20, and 60 kcal% for high-fat diets, respectively. Body weights were measured 3 times a week. The number of mice in each group was 9 (except n = 6 for female WT).

Micro-computed tomography (CT)

In-vivo micro-computed tomography imaging was performed on a MicroCAT II scanner (Siemens/Imtek, Inc., Knoxville, TN). Scans were acquired with the following settings: X-ray voltage was set at 55 kVp and anode current was 500 µA with a shutter speed of 500 milliseconds (ms). Scans were completed over 360° of rotation with 360 projections. The total time for each scan was 10 min. Images were acquired and reconstructed at 91 µm resolution. Raw images were reconstructed with Cone Beam Reconstruction Apparatus (COBRA) software (Exxim Computing Corporation, Pleasanton, CA). All images were calibrated to Hounsfield Units (HU) by scanning a water phantom with scan parameters identical to those used for imaging the mouse. Densities were calculated by scanning phantoms that had known densities of 1536.767mg/cc, 1227.121mg/cc, 1083.537mg/cc and 1057.299 mg/cc with the same scan parameters as described above. The density phantoms HU was then calculated using Amira 3.1 (Mercury Computer Systems, Inc., San Diego, CA). The bone, lung, WAT, BAT and lean body mass of the mice were then compared to the known phantoms mass, density and HU by using a trend formula in which known y's (density of phantoms) and known x's (HU of density phantoms) returns the y-values along that line for the array of new x's (selected mouse regions HU). In the regions of interest (ROI), the number of voxels and area were calculated by taking the dimensions of the scan 512 x 512 x 896 and multiplying by the

voxel size 0.091. This equates to the X and Y axis having a ROI of 4.659cm and the Z axis 8.154 cm.

Exercise testing

WT and KO male mice (22 weeks old) were subjected to treadmill exercise, as previously described [2]. For graded maximal treadmill exercise, mice were acclimated by running for 10 min at 10 m/min for 2 d and maximum exercise capacity determined by graded increase in treadmill speed (10, 12, 14, 16, 18 and 20 m/min for 2-5 min at each speed followed by 2 m/min increase every 5 min) on a 5% incline to exhaustion. The mice were continually monitored during the exercise regimen; if an animal became exhausted, the shock bars for that animal were turned off and the animal was allowed to rest at the back of the treadmill.

Whole body oxygen consumption

Oxygen consumption in intact mice was measured in WT and KO as previously described [3].The effect of the β3-selective agonist, CL316,243 (CL), was measured as follows (each mouse serving as its own control): At ~9 A.M. mice were placed into the calorimetry chambers (pre-warmed to 30°C) and baseline data were collected. After 3 h, CL was injected intraperitoneally (4, 30, or 200 µg/kg). After equilibration (1 h), data were collected for a 2-h period.

Oxygen consumption in eWAT and BAT

The Clark oxygen sensor electrode (DW1, Hansatech Instruments, Norfolk, UK) was mounted in a chamber according to the manufacturer's instructions and connected to a computer operated control unit to register cellular respiration (Oxygraph software, Hansatech). Prior to the experiment, the oxygen electrode was calibrated in Krebs Ringer HEPES (KRH) buffer (25 mM HEPES pH 7.5, 120 mM NaCl, 4.74 mM $CaCl_2$, 2 mM glucose, 200 µM adenosine, 1% fatty acid free BSA) at 37°C. A 2-point calibration was performed between the oxygen levels of air-saturated buffer and zero oxygen buffer. eWAT and interscapular BAT were excised from WT and age-matched KO mice (12-16 weeks old), and immediately placed in KRH buffer. The tissues were analyzed for oxygen consumption within 2 h after excision. KRH buffer (500 µl/experiment) was prewarmed to 37°C in the oxygraph chamber and the measurement was started by establishing a stable background. A piece of WAT (50±10 mg) or BAT (10±3 mg) was minced 30 times with a pair of scissors and thereafter added to the KRH buffer in the chamber. The samples were continuously stirred with a magnetic stirrer and the lid of the chamber was adjusted to the sample volume. The oxygen consumption, calculated (after subtraction of background) as O_2 consumption nmol/min/mg tissue, was measured during the first 6 min after addition of the tissue.

Isolation of adipocytes from eWAT

Adipocytes were isolated from eWAT by collagenase digestion as described previously [4]. Briefly, fat pads were removed, transferred into Krebs-Ringer phosphate HEPES buffer (KRH) (130 mM NaCl, 4.7 mM KCl, 1.24 mM $MgSO_4$, 2.5 mM $CaCl_2$, 1 mM HEPES, 2.5 mM NaH_2PO_4, 5 mM D-glucose, 3% BSA, and 200 nM adenosine, pH 7.4) at 37°C, and minced and digested with collagenase B (Sigma) (3.3 mg/ml) in KRH buffer (45 min, 37°C) in a shaking water bath (120 rpm). The fat cell suspension was filtered through 250-µm nylon mesh and centrifuged (10 sec, 1000 rpm). Adipocytes, collected from the top phase, were washed with KRH buffer (four times), resuspended in 5 volumes of KRH buffer, equilibrated (10 min, 37°C), and then used immediately for experiments.

Fatty acid oxidation (FAO) assay

For each experiment, adipocytes were prepared from eWAT of 2 WT and 2 KO mice (5 month old), and used for FAO studies and analyzed for DNA content. For FAO assays, stock solutions of palmitic acid bound to fatty acid-free bovine serum albumin (BSA) were prepared, and nonesterified fatty acid concentrations verified using the NEFA C kit (Wako Chemicals, Richmond, VA). Adipocyte suspensions (in duplicate) were incubated with BSA-bound palmitic acid (4.3 mol/L) and ^3H labeled palmitic acid ([9,10-^3H(N)], PerkinElmer Life Sciences, Boston, MA) (55.6 pmol/L) in 5 mM glucose, Krebs-Ringer HEPES albumin buffer (pH 7.4), containing 2.0 mg/ml fatty acid-free BSA at 37°C for 0, 30, 60, and 90 min, respectively, in a shaking water bath (80 rpm). At indicated time points, portions (0.2 ml) were added to a microtube that contained mineral oil (0.2 ml), and centrifuged (10,000 rpm, 2 minutes). The lower aqueous phase (0.1 ml) was added to a column containing 1 ml of resin (Bio-Rad AG1-X8, 200-400 mesh) that retained non-oxidized ^3H labeled palmitic acid, but allowed oxidized palmitate (in the form of ^3H_2O) to pass through [5]. The columns were eluted with 3 ml double-distilled H_2O, directly collected into a scintillation vial, and ^3H_2O production was quanitfied. Oxidized palmitic acid was calculated as follows: oxidized palmitic acid (pmol) = (sample dpm-blank dpm)/(total dpm-blank dpm) x total amount of palmitic acid (pmol) [5]. Adipocyte DNA content was quantified by fluorometry [6], using bis-benzamide and calf thymus polymerized DNA (Sigma) as standard. Results were expressed as pmol oxidized palmitic acid per µg adipocyte DNA.

Mitotracker staining and laser scanning confocal immunofluorescence

eWAT and/or interscapular BAT fat pads were removed and fixed for 16 h at room temperature in Formalin (10%) buffered in Phosphate (Electron Microscopy Sciences, Hatfield, PA), and

embedded in paraffin. Paraffin sections were dewaxed in xylene, and rehydrated through graded ethanol, Some sections were incubated with 500 nM MitoTracker Red chloromethyl-X-rosamine (CMXRos) or Mitotracker Green (MTG) (Molecular Probes, Eugene, OR) for 10 min at room temperature. Slides were washed, mounted, and observed with a Fluorescence microscope (Carl Zeiss, Thornwood, NY, 400x).

Other dewaxed/rehydrated paraffin sections were washed in PBS 3 x 5 min, and blocked and permeabilized in 10% donkey serum containing 0.05% Triton X100 for 6 h at 4°C. Slides were incubated in blocking buffer with primary anti-smooth muscle actin (SMA) or anti-CD31 antibodies (overnight, 4°C, and washed with PBS (3 x 5 min) before incubating in blocking buffer for 2 h with secondary antibodies (Alexa Fluor 488 or alexa fluor 594) (Molecular Probe). As controls, samples were also incubated with nonimmune IgG or with primary antibody incubated with blocking peptides prior to staining with secondary antibody. Slides were viewed with a Zeiss LSM510 laser scanning confocal microscope.

Mitochondria isolation and respiratory analysis

WT and KO eWAT and WT interscapular BAT were homogenized in mitochondria isolation buffer [250 mM sucrose, 20 mM HEPES, 1 mM EDTA, 1 mM EGTA, 1 mM DTT and protease inhibitor cocktail (Thermoscientific, Rockford, IL)], and centrifuged at 1000 xg for 10 min. The supernatant was then centrifuged at 18,000 xg for 30 min to produce a mitochondrial pellet. The pellet was rehomogenized and centrifuged at 77,000 xg for 1 h on a discontinuous sucrose gradient (25%, 35%, 45% sucrose). Material at the 25-35% interface was collected and designated as *Upper* and material at the 35-45% interface was designated as *Lower*. Both were diluted in mitochondria isolation buffer and finally centrifuged at 18,000 xg for 30 min to collect mitochondrial fractions. Mitochondrial respiration was measured using a Clark-type O_2 electrode (Instech Laboratories, Plymouth Meeting, PA) and O_2 monitor (Model 5300, YSI, Inc) as described previously [7]. Mitochondria (18,000 xg pellets) were resuspended in respiration buffer (pH 7.25) containing 120 mM KCl, 5 mM MOPS, 1 mM EGTA, 5 mM KH_2PO_4, and 0.2% BSA, and basal respiratory rates were calculated in the presence of 10 mM glutamate/2 mM malate, and 0.5 mM ADP. Uncoupled respiration was evaluated in the presence of 4 mM succinate and 1 µg/ml oligomycin with or without the UCP antagonist GDP (0.5 mM) as described previously [8]. Since mitochondrial contents are increased in KO eWAT, the respiration rate was normalized by the amount of mitochondrial protein determined using Bradford assay.

Electron microscopy (EM)

Mitochondrial fractions isolated from fresh tissues as described above were fixed by addition of 1x fixative (2% Glutaraldehyde in 0.1 M cacodylate buffer) and incubation at 4°C. Mitochondrial pellets were then processed by Electron Microscope Facility Image Analysis Laboratory, NCI-Frederick, NIH, MD.

Two-dimensional difference gel electrophoresis (DIGE) analysis

CyDye two-dimensional (2D) fluorescence difference gel electrophoresis (DIGE) was performed as described previously [9]. Briefly, KO and WT eWAT mitochondrial pellets were resuspended in lysis buffer containing 15 mM Tris-HCl, pH 8.5, 7 M urea, 2 M thiourea, and 4% CHAPS. Individual samples (50 µg) were labeled on Lys residues with Cy3 (WT) and Cy5 (KO) (GE Healthcare, Piscataway, NJ). A 50-µg internal standard consisting of equal protein amounts of all samples was labeled with Cy2. The labeled samples and internal standard were combined for DIGE electrophoresis. Unlabeled samples (500ug) were run on separate gels for spot picking. First dimension isoelectric focusing was carried out using IPG (immobilized pH gradient) strips (pH 3-10 non-linear) for a total of 63 kV·h (Ettan, IPGphor, GE Healthcare, Piscataway, NJ). The strips were then loaded onto an Ettan DALT-12 electrophoresis unit (GE Healthcare, Piscataway, NJ), and the proteins were separated on a 10-15% SDS-polyacrylamide gel (NextGen) at room temperature for 16 h under constant voltage (105 V). The Cy2 images were scanned at an excitation wavelength of 520/40 (maximal/bandwidth) using a blue laser while the Cy3 images were scanned with an excitation wavelength of 580/30 using a green laser. The Cy5 images were scanned using a 670/30 excitation wavelength and a red laser using the Typhoon 9400 Variable Mode Imager (GE Healthcare, Piscataway, NJ). The spot pick gels were stained with EZBlue gel staining reagent (Sigma) following manufacturer procedures. Image analysis for the differences between WT and KO mitochondrial proteins (e.g. KO/WT) was performed using Progenesis Discovery software (NonLinear Dynamics, Durham, NC). Spots of interest were matched to the spot pick gels using the software for protein identification.

Identification of eWAT mitochondrion proteome

For all protein identifications from 2D spot pick gels (Table S2), protein spots were picked with the Ettan Spot Handling Workstation (GE Healthcare, Piscataway, NJ). Protein identification was carried out with the 4700 Proteomics Analyzer (MALDI-TOF/TOF) instrument (Sciex, Framingham, MA) with reflector positive ion mode. For mass spectrometry (MS) analysis, an 800–4,000 mass-to-charge ratio (m/z) mass range was used with 1,500 shots per spectrum. Result-dependent analysis (RDA) was used for MS/MS selection. A maximum of six precursors per protein were selected, with a confidence interval (CI) percentage of 50 or higher and a minimum signal-to-noise ratio of 50. In

addition, a low-confidence investigation (peptides not matched to top proteins) was used to allow a maximum of five precursors per spot with minimum signal-to-noise ratio of 50 and selected for data-dependent MS/MS analysis. A 1-kV collision energy was used for collision-induced dissociation (CID), and 1,500 acquisitions were accumulated for each MS/MS spectrum. For both MS and MS/MS analysis, the default calibration was performed with 4700 mass standard peptide mix (Sciex, Framingham, MA) achieving a mass accuracy within 50 ppm. Internal calibration was used for all MS runs with trypsin autolysis peaks of 842.51 m/z, 1,045.56 m/z, and 2,211.11 m/z. When one or more of the trypsin peaks were not found within the mass tolerance of 0.1 m/z, default processing was used.

The peak lists were generated with GPS Explorer software using default parameters (version 3.0, Sciex, Framingham, MA). Mascot search engine was used (version 2.2, Matrix Science, Boston, MA) for peptide and protein identifications with the following search criteria: enzyme, trypsin; miscleavages, one; fixed modifications, cysteine carbamidomethylation; variable modifications, methionine oxidation; mass tolerance for precursor ions, 100 ppm; and mass tolerance for fragment ions, 0.5 Da. The SwissProt protein knowledgebase database was searched against, and MS peak filtering was set for all trypsin autolysis peaks. The species selected was *Mus musculus* (mouse), and the number of sequence entries searched in the *M. musculus* database. The acceptance criteria for protein identifications had to meet the following criteria; identification of two peptides or more with a MS/MS confidence interval (CI%) > 95%, molecular weight and pI had to match the position where the spot was picked on the 2D gel. The *P* value was chosen to reflect a 95% probability that the protein identification is correct.

Preparation of homogenates, crude and solubilized membrane fractions, and cytosolic fractions for partial purification (DEAE and gel filtration chromatography) and Western blotting

Fresh mouse eWAT was collected and homogenized (1/3, w/v) in Buffer A [50mM Hepes pH 7.4, 1mM EDTA, 1mM EGTA, 50 mM sucrose, 50mM NaCl, 1mM DTT, protease Inhibitor Cocktail and Phosphatase Inhibitor Cocktail (Thermoscientific, Rockford, IL)], using a Dounce glass homogenizer (20 strokes on ice), and centrifuged (500 xg, 15 min, 4°C).

To prepare total adipose tissue extracts (homogenates), pellets were resuspended in Buffer A, rehomogenized, and centrifuged (500 xg, 15 min, 4°C). Supernatants (500 xg) were pooled, sonicated (on ice, 20 pulses, 40% duty cycle, output scale 4) in buffer A containing 1% (v/v) Triton-X100, and incubated with rotation (4°C, 1 h) before centrifugation (15,000 xg, 20 min, 4°C). These supernatants (designated as total adipose tissue extracts or homogenates) were used for protein

measurements, PDE assays, or comparative protein expression analysis by Western immunoblotting (using samples of WT and KO eWAT homogenates).

In some experiments, initial homogenates were centrifuged (1,000 xg, 15 min, 4°C), and pellets were utilized for extraction of nuclear proteins as described [10]. Nuclear pellets were washed twice by resuspension in buffer A and centrifugation (1000 x*g,* 10 min, 4°C). Nuclei were then resuspended in buffer A containing 0.5 M NaCl and 1% Triton-X100, incubated with incubation/rotation (4°C,1 h), and centrifuged (10,000 x*g*, 10 min). These supernatants were designated as nuclear extracts and used for Western blotting. In some experiments, nuclear proteins were extracted using the Nuclei PURE Prep Nuclei Isolation Kit and CelLytic NuCLEAR Extraction Kit (Sigma), according to manufacturer's instructions.

To prepare total membrane and cytosol fractions, homogenates were briefly sonicated on ice and centrifuged (1,000 xg, 15 min, 4°C). Supernatants were centrifuged (100,000 xg, 1 h, 4°C). These pellets were defined as total membrane fractions, and the resulting supernatants as cytosol. Membrane pellets were homogenized (using a Dounce homogenizer) and sonicated (on ice, 20 pulses, 40% duty cycle, output scale 4) in buffer A containing 1% (v/v) Triton-X100, and, after incubation/rotation (4°C, 1 h), were centrifuged (15,000 xg, 20 min, 4°C). Solubilized membranes (15,000 xg supernatants) or cytosolic fractions were used for PDE assays or Western blotting or partially purified via DEAE Sephacel Fast Flow anion exchange (GE Healthcare) or gel filtration chromatography (FPKLC-superose 12; AKTA FPLC system; GE-Healthcare, Piscataway, NJ, USA). Equivalent amounts and volumes of solubilized membrane fractions, cytosolic fractions, and nuclear fractions (usually 30 µg/lane) or total adipose tissue homogenates were subjected to SDS-PAGE using Tris-Glycine Gels (Invitrogen). Separated proteins were transferred to nitrocellulose membranes (Invitrogen). The membranes were incubated (4°C, overnight) with blocking buffer containing 5% (w/v) NFDM (non-fat dry milk) in DPBS (Dulbecco's PBS), and then with the appropriate primary antibody in blocking buffer (usually for 2-4 h, but sometimes longer, depending on quality and sensitivity of the antibody). After incubation with primary antibody, membranes were washed in PBS (3 x 5 min), and incubated (2 h) with HRP (horseradish peroxidase)-labelled secondary antibodies (Pierce) and washed with PBS (3 x 5 min). Immunoreactive proteins (membranes) were incubated with SuperSignal® Westpico or Westfemto chemiluminescent reagents; signals were detected with an ImageQuant Imagereader LAS4000 (GE Healthcare). Band densitometry was measured with Multi Gauge V2.3 software and the resultant individual values of target homogenate or nuclear proteins were normalized by the values for β-actin or histone H3, respectively.

Measurement of AMPK activity

Fresh eWAT or 3T3-L1 adipocytes were collected and homogenized in buffer A containing 0.5 μM okadaic acid, and supplemented with 1% Triton X100. After centrifugation (4000 xg, 15 min, 4°C), supernatants were adjusted to 6% PEG 6000 and incubated (45 min, on ice). Following further centrifugation (18,000 xg, 15 min), pellets were resuspended in buffer A; protein concentration was adjusted to 0.4 mg/ml with Buffer A. PEG-precipitated protein (5 μl, 2 μg) was assayed in duplicate for AMPK activity. Reactions (50 μl) contained sample protein or positive control (25 mU AMPK activity, Catalog # 14-305, Upstate, Charlottesville, VA), 5 μl of 10X reaction buffer (400 mM HEPES, pH 7.4, 800 mM NaCl, 50 mM $MgCl_2$, 1 mM DTT), 10 μl of SAMS peptide (Upstate, 1 mg/ml), 5 μl of ATP working solution (1 μl of 10 mM ATP, 0.5 μl of [γ-^{32}P]ATP (5 μCi), and 3.5 μl of H_2O), and 25 μl of H_2O or 400 μM AMP, respectively. Solutions were finger-vortexed, then briefly spun down, and incubated (37°C, 15 min). Portions (20 μl) of reaction mixtures were spotted onto P81 Whatman paper (Upstate), which were washed 4 times with 1% phosphoric acid, dried, and counted to determine the amount of bound phosphorylated SAMS peptide. The difference in cpm between the presence and absence of AMP was calculated and converted to AMPK units (Unit/gram protein/minute) by normalization to activity of the positive control enzyme samples (AMPK, Catalog # 14-305, Upstate).

Isolation of RNA from cultured 3T3-L1 adipocytes

3T3-L1 fibroblasts were purchased from ATCC (Manassas VA) and propagated (37°C, 5% CO_2) in growth medium [DMEM high glucose medium (Invitrogen) with 10% fetal bovine serum (ATCC)]. After reaching confluence, fibroblasts were induced to differentiate by incubation with growth medium containing 0.5 mM 3-isobutyl-1-methyl-xanthine (Sigma), 1 μM dexamethasone (Sigma), and 10 μg/ml insulin (Sigma) for 3 days, at which time the medium was changed to growth medium containing 10 μg/ml insulin. 3T3-L1 adipocytes were routinely used for experiments on day 10-12 after initiation of differentiation. Total RNA was isolated using RNeasy Mini Kit (Qiagen, Chatsworth, CA); electrophoresis (1% agarose gel) confirmed RNA integrity. Total RNA was diluted to 10 ng/μl, and 100 ng of RNA were subjected (in duplicate) to Real-time quantitative RT-PCR on the HT7900 Sequence Detection System (Applied Biosystems) by using QuantiTect SYBR Green RT-PCR kit (Qiagen) according to manufacturer's protocols. The value of the target gene was normalized by that obtained from cyclophilin A, which served as the internal control,

siRNA knock-down of PDE3B in 3T3-L1 adipocytes

3T3-L1 fibroblasts (ATCC, Manassas, VA) were propagated (37°C, 5% CO_2) in DMEM high glucose medium (Invitrogen) with 10% fetal bovine serum (ATCC). After reaching confluence, fibroblasts were induced to differentiate as described above. Using DeliverX Plus siRNA transfection kits

(Panomics) according to the manufacturer's protocol [11], siRNA duplex oligonucleotides corresponding to murine (M) PDE3B mRNA (cat no. L-043781-00) (siPDE3B) were utilized to knockdown PDE3B in 3T3-L1 adipocytes. Non–targeting/scrambled RNA (cat no. D-001810-10) (Dharmacon) (scRNA) was used as a negative control. Specific PDE3B knock-down was confirmed via immunoblotting, PDE3 activity assays, and quantitative real-time RT-PCR.

cAMP PDE assay

Samples (usually 0.1 ml) were incubated (usually 10 min) at 30°C in a total volume of 0.3 ml containing 50 mM HEPES, pH 7.5, 8.3 mM $MgCl_2$, 0.1 mM EDTA, and 0.1 μM [^{3}H]-cAMP (25,000-35,000 cpm) as substrate. After dephosphorylation of [^{3}H]-5-AMP with *Crotalus atrox* venom (Sigma, St. Louis, MO), [^{3}H]-adenosine product was separated from [^{3}H]-cAMP substrate by ion-exchange chromatography (QAE-Sephadex A-25;GE Healthcare), and quantified by scintillation counting [12]. PDE3 activity is that portion of total PDE activity inhibited by 1.0 μM cilostamide, a specific PDE3 inhibitor with an IC_{50}, 17~80 nM [13].

DEAE partial purification of eWAT cytosolic fractions

To prepare total membrane and cytosol fractions, fresh mouse eWAT were collected, and homogenized (1/3, W/V) in Buffer A [50mM Hepes pH 7.4, 1mM EDTA, 1mM EGTA, 50 mM sucrose, 50mM NaCl, 1mM DTT, Protease Inhibitor Cocktail and Phosphatase Inhibitor Cocktail (Thermoscientific, Rockford, IL)], using a Dounce glass homogenizer (20 strokes on ice). Homogenates were briefly sonicated on ice and centrifuged (1,000 xg, 15 min, 4°C). Supernatants were centrifuged (100,000 xg, 1 h, 4°C). These pellets were defined as total membrane fractions, and the resulting supernatants as cytosol. Membrane pellets were homogenized (using a Dounce homogenizer) and sonicated (on ice, 20 pulses, 40% duty cycle, output scale 4) in buffer A containing 1% (v/v) Triton-X100. After incubation/rotation (4°C, 1 h), solubilized membrane proteins were prepared by centrifugation (15,000 xg, 20 min, 4°C).

For partial purification of PDE3 from cytosolic fractions of fresh eWAT, econo-pac polypropylene columns (1.5 x 12 cm, 20 ml bed volume) were packed with 4.0 ml DEAE Sephacel Fast Flow (GE-Healthcare) preequilibrated with buffer A. Cytosolic fractions (~50 mg) from WT or KO mice were passed 2-3 times through the DEAE columns (or incubated batch-wise for 1 h at 4°C). The DEAE columns were washed 3 times with buffer A (10 ml x 3). Fractions containing PDE activity were eluted with buffer A containing 500 mM NaCl (10 ml, passed twice through the column) and eluates were further concentrated via Centricon (10 kD cut off) (Millipore, Billerica, MA).

Gel filtration of eWAT cytosolic and solubilized membrane fractions

Solubilized membrane proteins (3mg protein, 1ml), and portions of partially purified and concentrated cytosolic fractions (after DEAE chromatography, 3 mg protein, 1 ml) were subjected to gel filtration chromatography on FPLC Superose-12 HR 10/30 columns (AKTA FPLC system, GE Healthcare), which were equilibrated and eluted with buffer A (without sucrose) containing 150 mM NaCl and 1% v/v Triton-X100. Portions of indicated fractions (0.5 ml) were used for immunoblotting and immunoprecipitations, and for assay of PDE3 activity. Eluted PDE3 activity accounted for 70-90% of the original PDE3 activity loaded onto the Superose-12 column. PDE activities are expressed as pmoles of cAMP hydrolyzed/min/mg.

Supplementary References

1 Ahmad, F. *et al.* Differential regulation of adipocyte PDE3B in distinct membrane compartments by insulin and the beta3-adrenergic receptor agonist CL316243: effects of caveolin-1 knockdown on formation/maintenance of macromolecular signalling complexes. *The Biochemical journal* **424**, 399-410, doi:10.1042/BJ20090842 (2009).

2 Fewell, J. G. *et al.* A treadmill exercise regimen for identifying cardiovascular phenotypes in transgenic mice. *Am J Physiol* **273**, H1595-1605 (1997).

3 Yu, S. *et al.* Paternal versus maternal transmission of a stimulatory G-protein alpha subunit knockout produces opposite effects on energy metabolism. *The Journal of clinical investigation* **105**, 615-623, doi:10.1172/JCI8437 (2000).

4 Choi, Y. H. *et al.* Alterations in regulation of energy homeostasis in cyclic nucleotide phosphodiesterase 3B-null mice. *The Journal of clinical investigation* **116**, 3240-3251, doi:10.1172/JCI24867 (2006).

5 Cha, B. S. *et al.* Peroxisome proliferator-activated receptor (PPAR) gamma and retinoid X receptor (RXR) agonists have complementary effects on glucose and lipid metabolism in human skeletal muscle. *Diabetologia* **44**, 444-452 (2001).

6 Downs, T. R. & Wilfinger, W. W. Fluorometric quantification of DNA in cells and tissue. *Analytical biochemistry* **131**, 538-547 (1983).

7 Lagranha, C. J., Deschamps, A., Aponte, A., Steenbergen, C. & Murphy, E. Sex differences in the phosphorylation of mitochondrial proteins result in reduced production of reactive oxygen species and cardioprotection in females. *Circulation research* **106**, 1681-1691, doi:10.1161/CIRCRESAHA.109.213645 (2010).

8 McLeod, C. J., Aziz, A., Hoyt, R. F., Jr., McCoy, J. P., Jr. & Sack, M. N. Uncoupling proteins 2 and 3 function in concert to augment tolerance to cardiac ischemia. *The Journal of biological chemistry* **280**, 33470-33476, doi:10.1074/jbc.M505258200 (2005).

9 Hoffert, J. D., van Balkom, B. W., Chou, C. L. & Knepper, M. A. Application of difference gel electrophoresis to the identification of inner medullary collecting duct proteins. *Am J Physiol Renal Physiol* **286**, F170-179, doi:10.1152/ajprenal.00223.2003 (2004).

10 Thuillier, P., Baillie, R., Sha, X. & Clarke, S. D. Cytosolic and nuclear distribution of PPARgamma2 in differentiating 3T3-L1 preadipocytes. *Journal of lipid research* **39**, 2329-2338 (1998).

11 Ahmad, F. *et al.* Insulin-induced formation of macromolecular complexes involved in activation of cyclic nucleotide phosphodiesterase 3B (PDE3B) and its interaction with PKB. *The Biochemical journal* **404**, 257-268, doi:10.1042/BJ20060960 (2007).

12 Kincaid, R. L. & Manganiello, V. C. Assay of cyclic nucleotide phosphodiesterase using radiolabeled and fluorescent substrates. *Methods in enzymology* **159**, 457-470 (1988).

13 Sudo, T. *et al.* Potent effects of novel anti-platelet aggregatory cilostamide analogues on recombinant cyclic nucleotide phosphodiesterase isozyme activity. *Biochemical pharmacology* **59**, 347-356 (2000).

Table S1. Primer sequences for real-time qPCR

Official Symbol	Left primer (5'-3')	Right primer (5'-3')
mAcad-l	gcttcagcctccactcagat	ggctatggcaccgatacact
mAcad-vl	tctgtccagagcctcaaggt	agcctcaatgcaccagctat
mAdrb1	atcgttctgctcatcgtggt	atgaagaggttggtgagcgt
mAdrb3	acaggaatgccactccaatc	aaggagacggaggaggagag
mBmp4	caatggagccattccgtagt	gggagccaatcttgaacaaa
mBmp7	tggtcatgagcttcgtcaac	tggaaagatcaaaccggaac
mCact	ggacgtgctcaagtctcgat	tcggatcagctctctcaaca
mCideA	ctcggctgtctcaatgtcaa	tccttaacacggccttgaac
mCox4	agaaggcgctgaaggagaa	ctggatgcggtacaactgaa
mCpt2	gctctaaggtatctggcagc	ctggtggacaggatgttgtg
mCtBP	ctgaccagagaagatctggag	atctgctctacactctggactcg
mDio2	tctgctcagtctgtggttgg	aggactccttgcaccatgac
mElovl3	ggtcctttctctttcttctcagc	gggagaagattaggatgcttcag
mGyk	tattttctgaacatggcctcct	ctcccaataaggcgcatataac
mLrp130	tctcctcgcaagtagtacctttg	gatctatgttcatcgacctcctg
mMyoD	gctctctctgctcctttgagac	agtagggaagtgtgcgtgctc
mNcoR	tataacgctgcttctctgtctcc	ttctgaacctggtcgtaggtag
mNrbf1	tgctgtgaaaggatctgacg	gccatagttcccttggatca
mp107	ctgtagcttcagccactcaaag	ctgggtatagtgttggcagaaag
mPde3b	ccaattcctggcttacctca	gcaatctgtccagaaccaag
mPpara	agaccttgtgtatggccgag	actggcagcagtggaagaat
mPgc-1a	ccgagaattcatggagcaat	gtgtgaggagggtcatcgtt
mPrdm16	gcagatctctgaagacttggg	aaggagtaggcaccttctttcac
mRb1	gcctcagccttccatactca	gaaggcgtgcacagagtgta
mResistin	caggacctgtatgctttaggatg	tgtccagtctatccttgcacac
mSirt1	catttatcagagttgccaccaa	accaacagccttaaaatctgga
mUcp1	aactgtacagcggtctgcct	taagccggctgagatcttgt
mSlc27a1	ctgggacttccgtggacct	tcttgcagacgatacgcagaa
mCited1	aaccttggagtgaaggatcgc	gtaggagagcctattggagatgt
mCD137	cgtgcagaactcctgtgataac	gtccacctatgctggagaagg
mHoxc9	gcagcaagcacaaagaggagaag	gcgtctggtacttggtgtaggg
mTbx1	ggcaggcagacgaatgttc	ttgtcatctacgggcacaaag
mShox2	tggaacaactcaacgagctggaga	ttcaaactggctagcggctcctat
mTmem26	accctgtcatcccacagag	tgtttggtggagtcctaaggtc
mMyh11	aagctgcggctagaggtca	ccctccctttgatggctgag
mPrune2	gctgaagaggagcgagaaga	ccccatagtatcctccgtga
mAdipsin	catgctcggccctacatgg	cacagagtcgtcatccgtcac
m18S	gatgtgaaggatgggaagtacag	cttcttggatacacccacagttc

Table S2. DIGE spot analysis

Spot Number†	Accession Number§	Fold Changes‡	Name	pI**	Mr¶	Total Ion Score	Total Ion C.I. %	Peptide Count
66	P48036	-16.71	Annexin A5	4.83	35.8	32	98.28	2
59	P09103	-13.61	Protein disulfide-isomerase	4.75	55.2	110	100	4
45	P63017	-10.73	Heat shock cognate 71 kDa protein	5.37	70.9	73	100	4
56	P00173	-7.77	Cytochrome b5	4.90	15.2	94	100	4
57	P08113	-7.61	Endoplasmin, Heat shock protein 90 kDa beta member 1, 94 kDa glucose-regulated protein	4.72	90.1	132	100	6
68	P07724	-5.27	Serum albumin	5.53	65.9	164	100	7
100'	P04117	-5.00	Fatty acid-binding protein	8.55	14.5	59	100	3
58	P20029	-4.60	78 kDa glucose-regulated protein	5.01	70.5	208	100	7
65	P07356	-4.51	Annexin A2	7.53	38.5	123	100	4
58'	P20029	-3.99	78 kDa glucose-regulated protein	5.01	70.5	110	100	5
44"	P38647	-3.72	Stress-70 protein	5.50	68.6	186	100	8
67	P14824	-3.34	Annexin A6	5.34	75.8	97	100	4
44'"	P38647	-3.05	Stress-70 protein	5.50	68.6	58	100	3
100	P04117	-2.37	Fatty acid-binding protein	8.55	14.5	56	99.99	3
33	Q9R257	-2.13	Heme-binding protein 1	5.18	21.1	146	100	5
89	Q921H8	-2.11	*3-ketoacyl-CoA thiolase A	8.63	41.2	364	100	9
95	P00507	-2.11	*Aspartate aminotransferase	8.97	44.5	48	99.94	3
55	Q8VCT4	-2.02	*Carboxylesterase 3	6.18	59.8	194	100	6
93	Q99MN9	-2.02	*Propionyl-CoA carboxylase beta chain	7.18	59.0	84	100	6
43	Q9D855	-1.67	Cytochrome b-c1 complex subunit 7	9.10	13.4	181	100	5
98	Q64521	-1.61	Glycerol-3-phosphate dehydrogenase	5.82	76.6	140	100	6
77	P56574	-1.53	Isocitrate dehydrogenase [NADP]	8.49	46.6	47	99.94	2
9'	Q8BH95	-1.49	Enoyl-CoA hydratase	7.78	28.5	155	100	3
62	P10719	-1.41	ATP synthase subunit beta	4.95	51.7	637	100	12
14'	Q9DCW4	-1.40	Electron transfer flavoprotein subunit beta	8.29	27.5	369	100	8
39	P09671	-1.36	Superoxide dismutase [Mn]	7.30	22.2	127	100	4
38	Q9QZA0	-1.33	Carbonic anhydrase 5B	5.89	32.7	81	100	4
35'	Q5XIH7	-1.26	Prohibitin-2	9.83	33.3	223	100	6
37	Q9DCM2	-1.25	Glutathione S-transferase kappa 1	8.97	25.6	56	100	3
64	Q9DCX2	-1.23	ATP synthase D chain	5.53	18.6	141	100	3
61	Q03265	-1.23	ATP synthase subunit alpha	8.28	55.3	730	100	13
86	O08756	-1.22	3-hydroxyacyl-CoA dehydrogenase type-2	8.56	27.3	154	100	4
38'	Q9QZA0	-1.21	Carbonic anhydrase 5B	5.89	32.7	66	100	2
99	P10860	-1.18	Glutamate dehydrogenase 1	6.71	55.9	326	100	11
84	P16332	-1.18	Methylmalonyl-CoA mutase	6.08	79.4	268	100	5
44	Q9CR68	-1.15	*Cytochrome b-c1 complex subunit Rieske	11.61	7.9	258	100	6
30	P29410	-1.15	*Adenylate kinase isoenzyme 2	6.36	26.2	146	100	5
36	P67779	-1.14	Prohibitin	5.57	29.8	500	100	6
19"	P47738	-1.11	Aldehyde dehydrogenase	6.05	54.4	201	100	8
31	Q8K2B3	-1.10	Succinate dehydrogenase [ubiquinone] flavoprotein subunit	6.32	68.0	135	100	7
101"	Q99MR8	-1.07	Methylcrotonoyl-CoA carboxylase subunit alpha	6.68	74.4	96	100	5
26	Q8K3J1	-1.07	NADH dehydrogenase [ubiquinone] iron-sulfur protein 8	5.13	20.4	110	100	4
32	Q9CQA3	-1.07	Succinate dehydrogenase [ubiquinone] iron-sulfur subunit	8.69	28.8	140	100	5
80	P49432	-1.07	Pyruvate dehydrogenase E1 component subunit beta	5.29	35.8	197	100	6
103	Q8QZS1	-1.06	3-hydroxyisobutyryl-CoA hydrolase	6.24	39.2	113	100	4
60	P24270	-1.06	Catalase	7.72	59.6	373	100	13
42	Q9DB77	-1.05	Cytochrome b-c1 complex subunit 2	8.99	46.6	443	100	10
69"	P80299	-1.05	Epoxide hydrolase 2	5.86	62.3	42	99.89	2
92	Q8QZS1	-1.03	3-hydroxyisobutyryl-CoA hydrolase	6.24	39.2	113	100	4
71'	Q9Z2I9	-1.00	Succinyl-CoA ligase [ADP-forming] beta-chain	5.33	44.4	139	100	5
51	P11240	+1.01	Cytochrome c oxidase subunit 5A	5.01	12.4	222	100	5
46	P63038	+1.02	60 kDa heat shock protein	5.35	57.9	575	100	10
63	Q9DB20	+1.03	ATP synthase subunit O	9.80	21.0	206	100	10
19'	P47738	+1.03	Aldehyde dehydrogenase	6.05	54.4	334	100	12
96	Q02253	+1.03	Methylmalonate-semialdehyde dehydrogenase [acylating]	7.54	54.5	212	100	6
16	Q9DCS3	+1.06	Trans-2-enoyl-CoA reductase	8.66	34.5	149	100	5
85	O08749	+1.06	Dihydrolipoyl dehydrogenase	6.43	50.2	268	100	7
87	Q9JLZ3	+1.06	Methylglutaconyl-CoA hydratase	9.03	29.2	145	100	4
3	Q9DBL1	+1.06	Short/branched chain specific acyl-CoA dehydrogenase	6.06	44.0	118	100	5
21	Q99LC3	+1.07	NADH dehydrogenase [ubiquinone] 1 alpha subcomplex subunit 10	5.96	36.9	49	99.95	3
84'	P16332	+1.07	Methylmalonyl-CoA mutase	6.08	79.4	144	100	5
25	P52503	+1.07	NADH dehydrogenase [ubiquinone] iron-sulfur protein 6	6.64	10.8	92	100	3
28	Q8BFR5	+1.07	Elongation factor Tu	6.20	45.0	100	100	3
19	P47738	+1.08	Aldehyde dehydrogenase	6.05	54.4	90	100	3
35	Q5XIH7	+1.09	Prohibitin-2	9.83	33.3	321	100	6
21'	Q99LC3	+1.10	NADH dehydrogenase [ubiquinone] 1 alpha subcomplex subunit 10	5.96	36.9	55	99.99	4
23	Q91WD5	+1.11	NADH dehydrogenase [ubiquinone] iron-sulfur protein 2	5.86	49.2	124	100	4
44	P38647	+1.13	Stress-70 protein	5.50	68.6	695	100	14
73	P97807	+1.13	Fumarate hydratase	7.88	49.9	436	100	12
91'	O35855	+1.14	Branched-chain-amino-acid aminotransferase	7.70	41.2	121	100	2
74	Q99NA5	+1.14	Isocitrate dehydrogenase [NAD] subunit alpha	5.72	36.7	99	100	3
19'"	P47738	+1.14	Aldehyde dehydrogenase	6.05	54.4	126	100	4
17'	P11960	+1.15	2-oxoisovalerate dehydrogenase subunit alpha	5.93	45.6	199	100	8
19""	P47738	+1.16	Aldehyde dehydrogenase	6.05	54.4	528	100	13

(**Table S2. continued**)

17	P11960	+1.16	2-oxoisovalerate dehydrogenase subunit alpha	5.93	45.6	67	100	4
41	Q9CZ13	+1.16	Cytochrome b-c1 complex subunit 1	5.28	49.2	265	100	8
13'''	P13803	+1.17	Electron transfer flavoprotein subunit alpha	8.62	35.3	227	100	6
24	Q9DCT2	+1.18	NADH dehydrogenase [ubiquinone] iron-sulfur protein 3	5.45	26.5	103	100	5
60'	P04762	+1.18	Catalase	7.15	59.6	96	100	3
20	O88696	+1.19	Putative ATP-dependent Clp protease proteolytic subunit	7.05	30.1	48	99.95	4
52	P19536	+1.21	Cytochrome c oxidase subunit 5B	5.74	10.7	190	100	4
79	Q8VHF5	+1.22	Citrate synthase	7.80	49.2	190	100	5
13''	P13803	+1.23	Electron transfer flavoprotein subunit alpha	8.62	35.3	246	100	5
10	O35459	+1.23	Delta(3,5)-Delta(2,4)-dienoyl-CoA isomerase	7.60	36.4	211	100	5
22	Q66HF1	+1.24	NADH-ubiquinone oxidoreductase 75 kDa subunit	5.28	76.9	388	100	11
81	Q01205	+1.25	Dihydrolipoyllysine-residue succinyltransferase component of 2-oxoglutarate dehydrogenase complex	5.89	41.5	366	100	9
11	Q9CQ62	+1.25	2,4-dienoyl-CoA reductase	8.78	32.5	29	95.88	2
9	Q8BH95	+1.26	Enoyl-CoA hydratase	7.78	28.5	246	100	6
90	Q8QZT1	+1.26	*Acetyl-CoA acetyltransferase	8.81	41.4	283	100	7
94	Q8BWT1	+1.26	*3-ketoacyl-CoA thiolase	8.33	41.9	81	100	3
53	Q9R0H0	+1.26	Acyl-coenzyme A oxidase 1, peroxisomal	8.64	74.6	91	100	5
14	Q9DCW4	+1.29	Electron transfer flavoprotein subunit beta	8.29	27.5	369	100	8
13	P13803	+1.30	Electron transfer flavoprotein subunit alpha	8.62	35.3	217	100	5
13'	P13803	+1.33	Electron transfer flavoprotein subunit alpha	8.62	35.3	331	100	8
85'	O08749	+1.33	Dihydrolipoyl dehydrogenase	6.43	50.2	96	100	5
82	Q05920	+1.33	Pyruvate carboxylase	6.05	127.4	367	100	14
46''	P63038	+1.34	60 kDa heat shock protein	5.35	57.9	101	100	4
75	Q68FX0	+1.35	Isocitrate dehydrogenase [NAD] subunit beta	7.82	38.8	380	100	7
60''	P24270	+1.35	*Catalase	7.72	59.6	159	100	7
97	Q3ULD5	+1.35	*Methylcrotonoyl-CoA carboxylase beta chain	8.20	61.9	82	100	5
91	O35855	+1.36	Branched-chain-amino-acid aminotransferase	7.70	41.2	151	100	5
40	Q9CQN1	+1.37	Heat shock protein 75 kDa	6.25	0.1	233	100	6
27	Q8CGK3	+1.39	Lon protease homolog	5.69	98.9	180	100	6
79'	Q8VHF5	+1.39	Citrate synthase	7.80	49.2	55	100	3
81'	Q01205	+1.42	Dihydrolipoyllysine-residue succinyltransferase component of 2-oxoglutarate dehydrogenase complex	5.89	41.5	312	100	10
73'	P97807	+1.43	Fumarate hydratase	7.88	49.9	169	100	5
78	P04636	+1.44	Malate dehydrogenase	8.55	33.2	564	100	12
78'	P04636	+1.44	Malate dehydrogenase	8.55	33.2	325	100	6
12	O55137	+1.45	*Acyl-coenzyme A thioesterase 1	6.12	46.1	134	100	4
15	O55171	+1.45	*Acyl-coenzyme A thioesterase 2	6.30	45.1	94	100	3
44'	P38647	+1.48	Stress-70 protein	5.50	68.6	407	100	10
5	P45952	+1.50	Medium-chain specific acyl-CoA dehydrogenase	7.69	43.6	412	100	10
1	Q99JY0	+1.50	Trifunctional enzyme subunit beta	9.24	47.6	363	100	11
4	Q07417	+1.50	Short-chain specific acyl-CoA dehydrogenase	7.12	42.2	243	100	9
71	Q9Z2I9	+1.51	Succinyl-CoA ligase [ADP-forming] beta-chain	5.33	44.4	288	100	8
4'	Q07417	+1.54	Short-chain specific acyl-CoA dehydrogenase	7.12	42.2	27	95.14	2
101'	Q99MR8	+1.55	Methylcrotonoyl-CoA carboxylase subunit alpha	6.68	74.4	245	100	6
82'	P52873	+1.60	Pyruvate carboxylase	6.13	127.5	568	100	19
29	Q9CYW4	+1.65	Haloacid dehalogenase-like hydrolase domain-containing protein 3	6.31	28.0	90	100	2
46'	P63038	+1.66	60 kDa heat shock protein	5.35	57.9	267	100	9
83	Q8BMF4	+1.66	Dihydrolipoyllysine-residue acetyltransferase component of pyruvate dehydrogenase complex	5.70	58.8	217	100	7
2	Q64428	+1.72	*Trifunctional enzyme subunit alpha	8.95	78.6	138	100	4
54	P51660	+1.72	*Peroxisomal multifunctional enzyme type 2	8.77	79.4	154	100	6
6'	P15650	+1.73	Long-chain specific acyl-CoA dehydrogenase	6.26	44.7	287	100	6
6	P51174	+1.73	Long-chain specific acyl-CoA dehydrogenase	6.50	44.6	318	100	7
101	Q99MR8	+1.76	Methylcrotonoyl-CoA carboxylase subunit alpha	6.68	74.4	135	100	5
72	Q9ER34	+1.81	Aconitate hydratase	7.15	82.5	409	100	10
2'	Q64428	+1.84	Trifunctional enzyme subunit alpha	8.95	78.6	62	100	3
102	Q8CHT0	+1.93	Delta-1-pyrroline-5-carboxylate dehydrogenase	7.70	59.1	30	98.38	3
72'	Q99KI0	+2.08	Aconitate hydratase	7.40	82.5	283	100	8
72''	Q99KI0	+2.12	Aconitate hydratase	7.40	82.5	186	100	7
8	P52825	+2.15	Carnitine O-palmitoyltransferase 2	7.95	71.1	207	100	6
7	P50544	+2.16	Very long-chain specific acyl-CoA dehydrogenase	7.72	66.3	224	100	8
8'	P52825	+2.16	Carnitine O-palmitoyltransferase 2	7.95	71.1	109	100	5
8''	P52825	+2.18	Carnitine O-palmitoyltransferase 2	7.95	71.1	86	100	3
69'	P34914	+2.37	Epoxide hydrolase 2	5.85	62.5	73	100	4
69	P34914	+2.49	Epoxide hydrolase 2	5.85	62.5	258	100	6
7'	P50544	+2.70	Very long-chain specific acyl-CoA dehydrogenase	7.72	66.3	66	100	3
8'''	P52825	+2.88	Carnitine O-palmitoyltransferase 2	7.95	71.1	59	100	3
41'	Q9CZ13	+4.58	Cytochrome b-c1 complex subunit 1	5.28	49.2	153	100	5
18	P52196	+6.48	Thiosulfate sulfurtransferase	7.82	33.3	108	100	5

†Spot numbers are indicated in Figure S4.

§Accession number for Swiss-Plot protein database.

‡'+' and '-' indicate the factor increase or decrease in spot intensity of Pde3B-/- mice adipose tissue mitochondria proteome relative to Wt mice.

*Two proteins are identified from the same spot

**Isoelectric points.

¶Molecular weights (Mr x 10-3).

(**Table S2. continued**)

The eWAT mitochodrial proteome (total 145 protein spots) was identified by MALDI-TOF MS/MS. Relative differences in expression of MS/MS-identified proteins in eWAT mitochondria were based on image analysis of Cy3/Cy5 (KO/WT) fluorescence in DIGE gels (n=3) and expressed as Fold Changes (KO/WT) as described in *SI Materials and Methods*.

Supplementary Fig. 1

A

Male

	WT	HE	KO	WT	HE	KO	WT	HE	KO

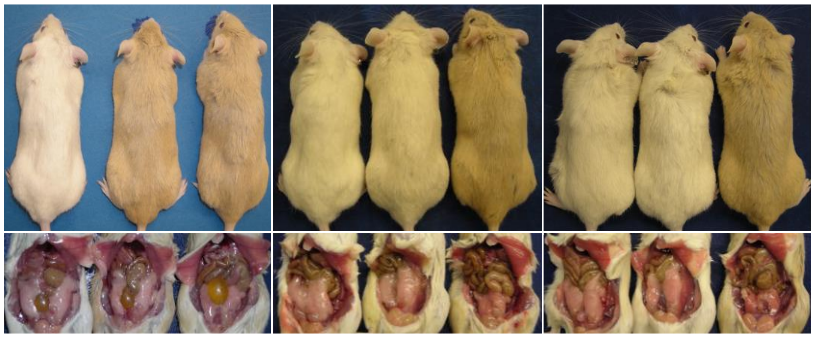

	WT	HE	KO	WT	HE	KO	WT	HE	KO
**Body Weight (g)**	37.2	35.5	41.2	34.2	35.2	40.9	27.6	29.7	31.1
**eWAT Weight (g)**	0.52	0.36	0.31	0.53	0.32	0.19	0.55	0.34	0.24
**% Fat (eWAT/body)**	1.40	1.01	0.75	1.55	0.91	0.46	2.00	1.15	0.77

B

Female

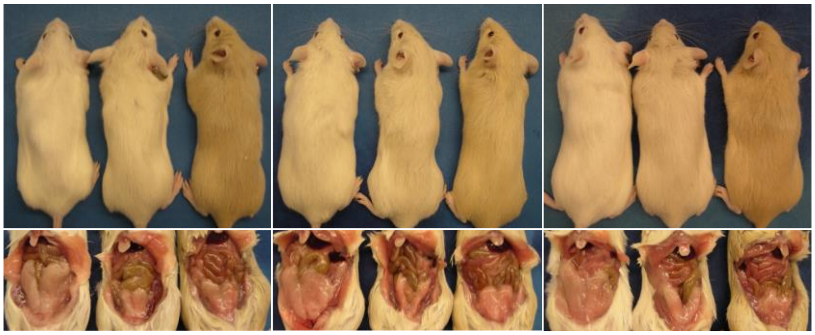

**Body Weight (g)**	28.5	23.9	28.2	27.0	23.3	24.2	21.5	22.5	23.3
**eWAT Weight (g)**	0.84	0.16	0.16	0.88	0.34	0.25	0.56	0.21	0.14
**% Fat (eWAT/body)**	2.95	0.67	0.57	3.26	1.46	1.03	2.61	0.93	0.60

C

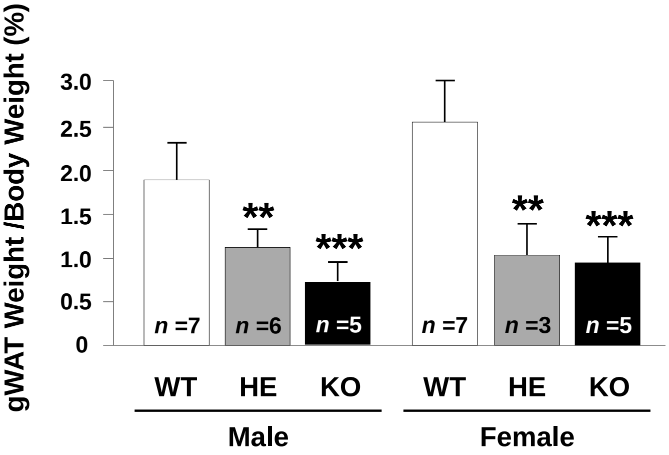

Figure S1. Smaller gonadal fat pads in PDE3B KO mice compared to their littermates
Representative photos of 6 groups of littermates, (*A*) male (7-10 months old) and (*B*) female (4-6 months old) mice, showing differences in coat color and smaller gonadal fat pads in KO mice. WT: wild type; HE: heterozygous ($PDE3B^{+/-}$); KO: homozygous ($PDE3B^{-/-}$). gWAT: gonadal white adipose tissue. (*C*) Percentages of male and female gWAT weight relative to body weight of male and female WT, HE, and KO littermates, housed at 1-2 mice per cage. Males (7-10 months): WT, 1.9 ± 0.42%; HE, 1.1 ± 0.20% ($^{**}p<0.01$ *vs.* WT); KO, 0.7 ± 0.21% ($^{***}p<0.001$ *vs.* WT). Females (4-6 months): WT, 2.6 ± 0.52%; HE, 1.0 ± 0.40% ($^{**}p<0.01$ *vs.* WT); KO, 0.9 ± 0.33% ($^{***}p<0.001$ *vs.* WT).

Supplementary Fig. 2

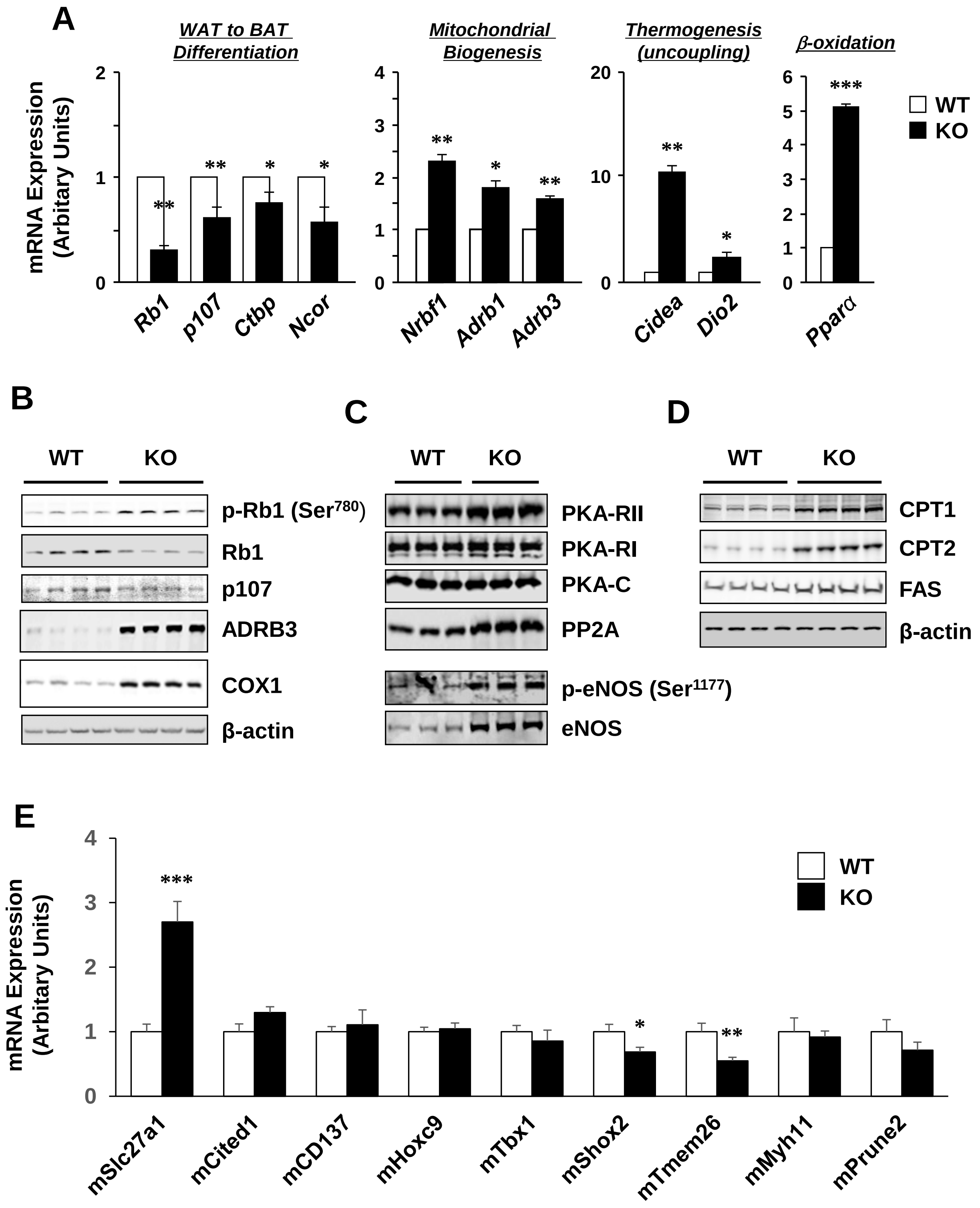

Figure S2. Gene and protein expression profiles related to WAT-to-beige phenotypic conversion: mitochondrial biogenesis, thermogenesis, and β-oxidation

(A) Real-time quantitative PCR (RT-qPCR) was performed as described in *SI Materials and Methods*. Primer sequences are listed in *Table S1*. (B-D) Protein expression was determined by Western blotting of WT and KO eWAT homogenates (30 μg). (E) RT-qPCR for beige adipocyte markers were performed. Primer sequences are listed in *Table S1*. Relative gene expression was normalized to *Adipsin* mRNA level. Data are presented as mean of arbitrary units ± SEM (n=5-11), relative to WT taken as 1; $^{*}p<0.05$; $^{**}p<0.01$; $^{***}p<0.001$ *vs.* WT, age-matched males.

Supplementary Fig. 3

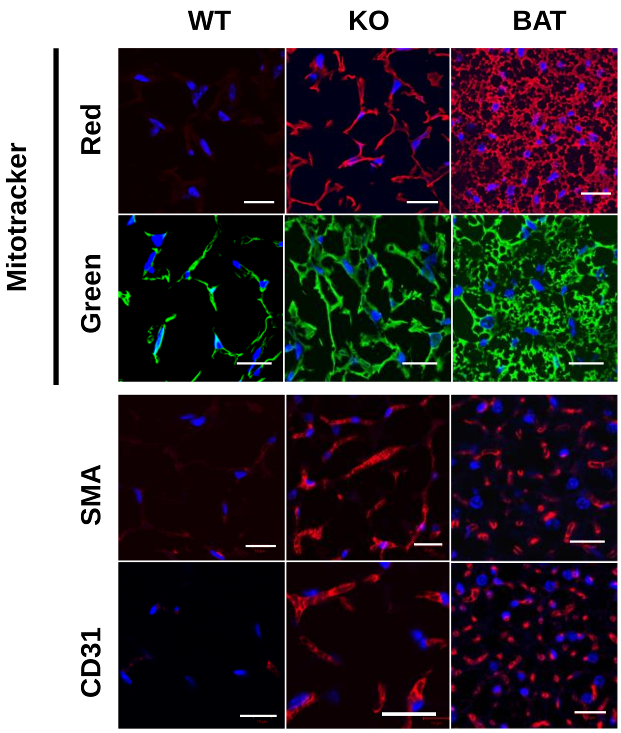

Figure S3. Increased mitochondrial density in PDE3B KO eWAT
Confocal microscopy. eWAT from WT and KO littermates, and interscapular BAT from WT mice were stained with mitochondrial and vascular markers. *Upper panels*: Mitotracker Red chloromethyl-X-rosamine (CMXRos) is a lipophilic cationic dye and concentrates inside mitochondria due to their negative mitochondrial membrane potential (MMP). Mitotracker Green (MTG) has been used as a measure of mitochondrial mass regardless of MMP. *Lower panels*: Tissue sections were stained with anti-smooth muscle actin (*SMA*, angiogenic markers) antibodies, anti-CD31 (endothelial cell marker) antibodies, and DAPI (nuclear staining), as described in *SI Materials and Methods*. Bars=10 µm.

Supplementary Fig. 4

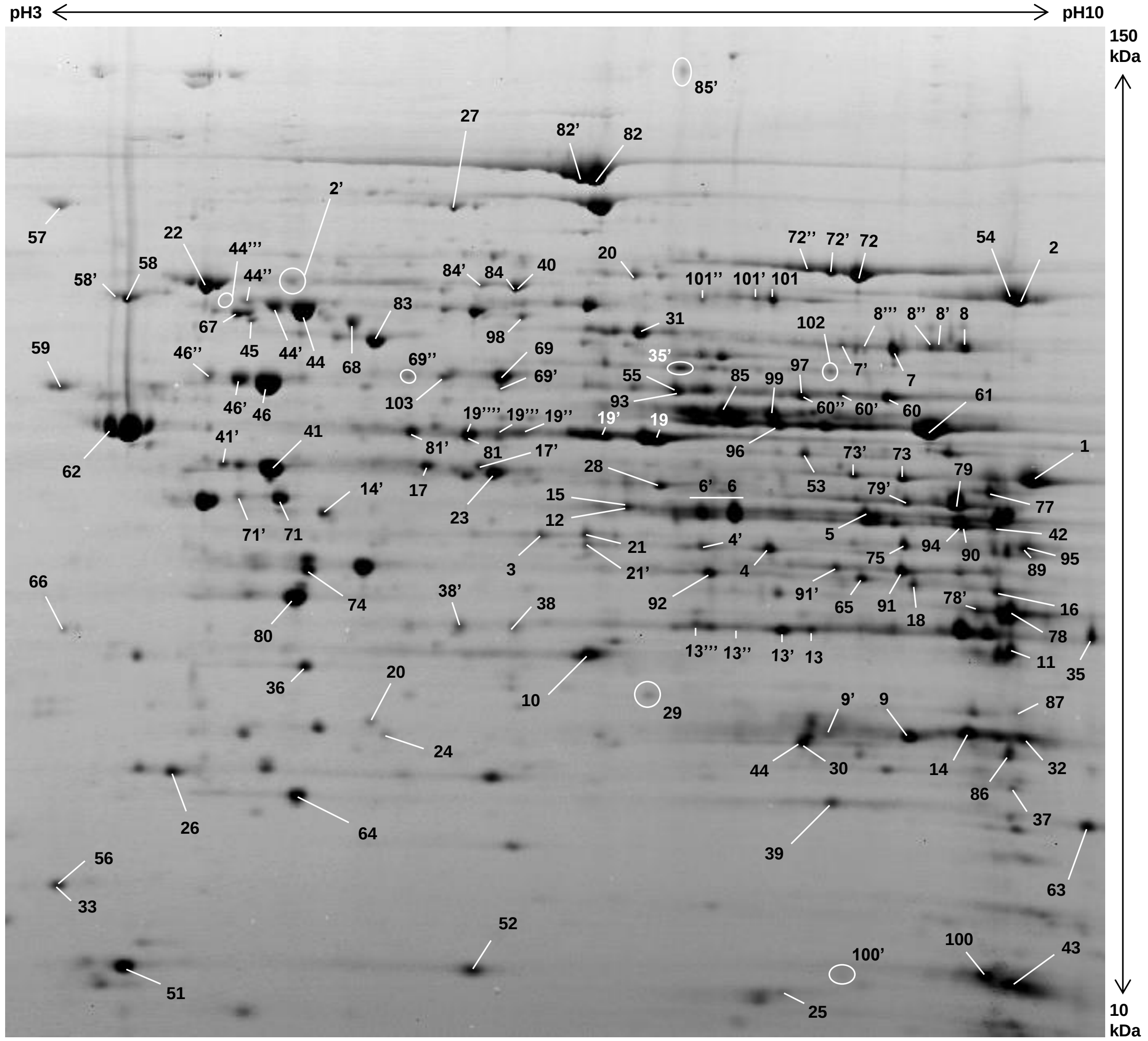

Figure S4. Mouse eWAT mitochondrial proteome
WT and KO eWAT mitochondrial preparations, labeled with cyanine dyes (Cy3 green for WT, Cy5 red for KO), were combined and analyzed by two-dimensional difference gel electrophoresis (DIGE) as described in *SI Materials and Methods*. This image is a gray scale of a coomassie blue-stained DIGE gel, and is representative of three independent gels. Spot information is listed in Table S2.

Supplementary Fig. 5

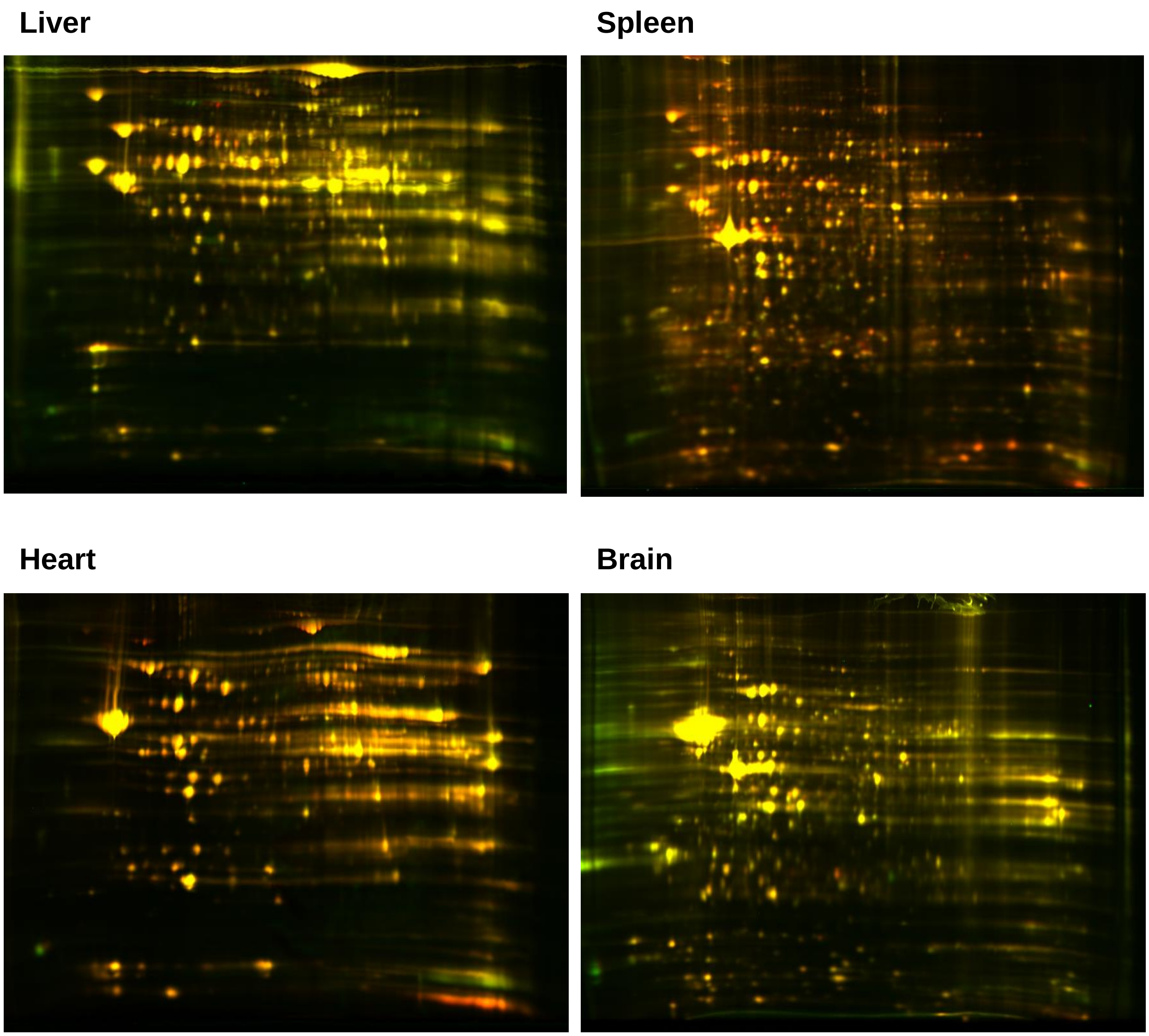

Figure S5. Mitochondrial proteome of mouse liver, spleen, heart and brain
Mitochondrial preparations from the indicated tissues of WT and KO mice, labeled with cyanine dyes (Cy3 green for WT, Cy5 red for KO), were combined and analyzed by DIGE as described in *SI Materials and Methods*.

Supplementary Fig. 6

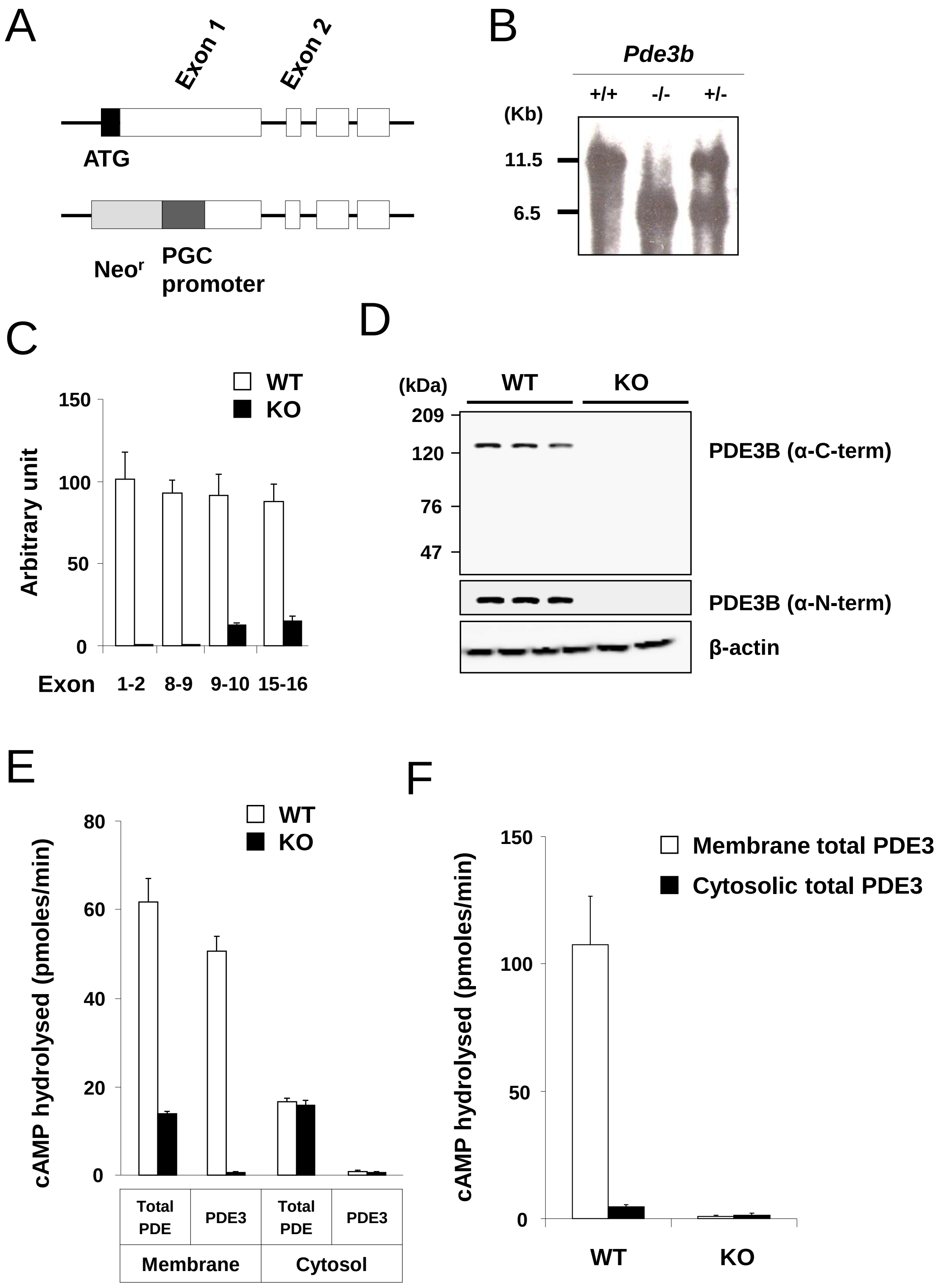

Figure S6. Targeted disruption of Pde3b gene

(A) Quantitation of *Pde3b* mRNAs in WT and PDE3B KO eWAT by real-time qRT-PCR, using boundary-specific primers for exons 1 and 2, 8 and 9, 9 and10, 15 and 16. Data were normalized to the quantity of *Pde3b* mRNA in WT mice measured using primers that amplified exon 1-2 region, taken as 100 AU. Values represent mean ± SD (*n*=3 of each genotype, duplicate assays). Data were similar in 2 other groups of WT and KO mice. Primers for amplification of different *Pde3b* exons are as follows: Exon1, 5'-aag cgc agc cgg tta cta t-3'; Exon2, 5'-caa ctc cat ttc cac ctc ca-3'; Exon8, 5'-aag agg cac agc aac caa at-3'; Exon9, 5'-gaa tcc ttc ctg att ttt ctc c-3'; Exon9, 5'-atg gga gaa aaa tca gga agg-3'; Exon10, 5'-gtg ata tgg aat gtc ccg gta g-3'; Exon15, 5'-ggt gaa gaa tta gat tca gat gat ga-3'; and Exon16, 5'-ttc ttc ttc tat gat ttc ctt cca-3'.

(B) Western blot of eWAT lysates (30 μg protein in each lane), prepared from WT and PDE3B KO mice (5 months old female). Results are representative of four experiments. Immunoblotting was performed using a monoclonal anti-β-actin antibody, and affinity-purified rabbit antibodies (Lofstrand Labs Ltd.) against N-terminal (3B N-T, RKDER ERDTP AMRSP PP, aa 2-18) or C-terminal (3B C-T, NASLP QADEI QVIEE ADEEE, aa 1076-1095) sequences of PDE3B. (*C and D*) Cytosol and solubilized membrane fractions were prepared from WT and KO eWAT (6 months old mice) as described above.

(C) Specific PDE activities (pmol cAMP hydrolyzed/mg protein/min), in WT and KO eWAT solubilized membrane and cytosolic fractions, were measured as described above, and presented as total PDE and PDE3 activities/mg protein/min. PDE3 activity is that portion of total PDE activity inhibited by 1.0 μM cilostamide, a specific PDE3 inhibitor. Data are means ± SD, duplicate assays, *n*=4 (WT), *n*=5 (KO) mice.

(D) Total membrane and cytosolic PDE3 activities were also calculated based on the recovery of total proteins from WT and KO eWAT. Recovery of total protein from WT eWAT membrane (2.1 ± 0.5 mg) and cytosol (4.9 ± 0.8 mg) fractions, and PDE3B KO eWAT membrane (3.2 ± 0.9) and cytosol (8.3 ± 1.7 mg) fractions indicated. Total protein recovery was increased ~40% in each PDE3B KO eWAT. Data are means ± SD, duplicate assays, *n*= 4 (WT), *n*=5 (KO) mice.

Supplementary Fig. 7

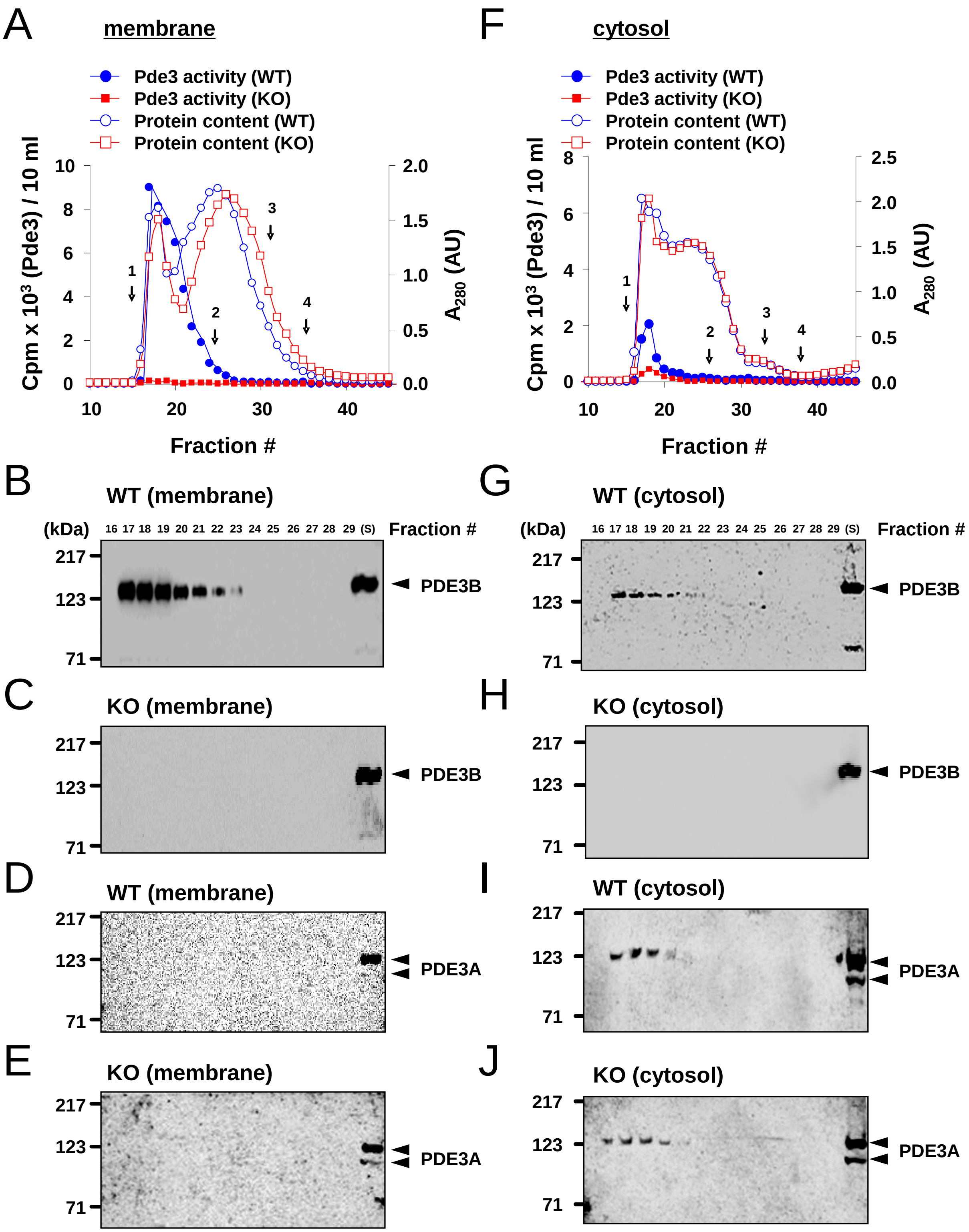

Figure S7. Gel filtration chromatography of solubilized eWAT membrane fractions and partially purified cytosolic fractions (**after DEAE chromatography**)

Solubilized eWAT membranes (3 mg protein) and partially purified cytosolic fractions (after DEAE columns, 3 mg), were prepared as described above, and subjected to gel filtration chromatography (FPLC-Superose 12, AKTA FPLC System, GE-Healthcare). *Left Panel*, membranes, and *right Panel*, cytosol. Protein content (AU 280 nm) (○, □) and PDE3 activity (PDE3 cpm/10 μl) (●, ■) were measured in indicated column fractions from WT (○, ●) and KO (□, ■) eWAT membranes (*A*), and cytosol (*F*); in this experiment, ~90% of the applied PDE3 activity from membrane and cytsosolic fractions was recovered in indicated fractions. Molecular weight standards: 1, thyroglobulin; 2, γ-globulin; 3, ovalbumin; 4, myoglobin. (*B*, *C*, *G*, and *H*) Western blots of (*S*) recombinant PDE3B (2 pmol PDE3 enzyme activity) as positive control (◄), and indicated fractions (20 μl) from (*B* and *C*) WT and KO membranes, and from (*G* and *H*) WT and KO cytosol were reacted with rabbit anti-PDE3B-CT antibody. (*D*, *E*, *I*, and *J*); Western blots of (*S*) lung homogenates (0.5 pmoles PDE3 activity) used as a positive control for mouse PDE3A (◄), and indicated eWAT fractions (20 μl) from (*D* and *E*) WT and KO membranes; and from (*I* and *J*) WT and KO cytosol were immunoblotted with rabbit anti-PDE3A-CT antibody. These results indicate that residual PDE3 activity in KO eWAT fractions (Fig. S6E and F and Fig. S7A and F) can most likely be accounted for by the presence of PDE3A (Fig. S7I and J).

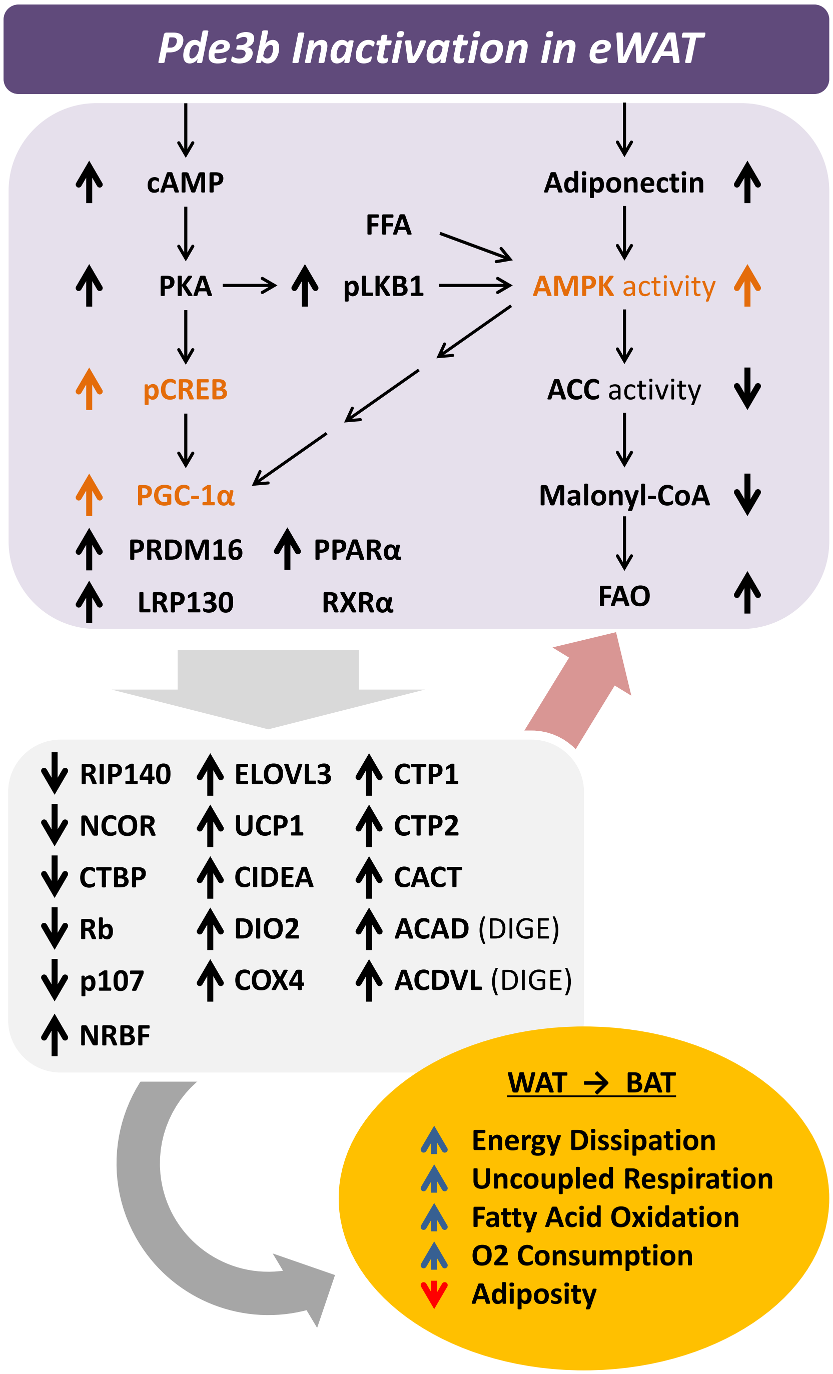

Figure S8. cAMP-dependent regulation of browning of eWAT, energy dissipation lipid metabolism, and inflammation in PDE3B KO Mice

PDE3B deletion may result in an increase of a special pool of compartmentalized cAMP, leading to activation of cAMP/PKA and AMPK signaling pathways, the integration of which triggered transcriptional regulation of expression of a number of genes crucial for development of the beige phenotype (PGC-1α PRDM-16, PPARα, SIRT3), and inducing pivotal genes for respiratory uncoupling (*e.g.* UCP1), mitochondrial biogenesis (*e.g.* ELOVL3, CIDEA, DIO2) and

fatty acid oxidation (*e.g.* CPT1, CPT2, CACT, ACAD), and decreased inflammatory markers (e.g., TRPV4). PDE3B deletion also increased the plasma level of adiponectin which contributed to activation of AMPK, which, in turn, led to a decrease in ACC activity and malonyl-CoA production, consequently increasing β-oxidation of fatty acids. Thus, in KO eWAT, cAMP-dependent and AMPK pathways play an important role in the regulation of the beige phenotype, energy homeostasis, FAO, lipid metabolism, and inflammation. Except for FFA, malonyl-CoA, and RXRa, all other genes and proteins listed were analyzed by RT-qPCR, Western blotting, or enzymatic activity.
